# Complex compositional and metabolic response of river sediment microbiomes to multiple anthropogenic stressors

**DOI:** 10.1093/ismeco/ycaf079

**Published:** 2025-05-17

**Authors:** Tom L Stach, Aman Deep, Iris Madge Pimentel, Dominik Buchner, Mikayla A Borton, André R Soares, Jörn Starke, Till L V Bornemann, Philipp M Rehsen, Ken L Dreger, Jens Boenigk, Matthijs Vos, Florian Leese, Daniela Beisser, Alexander J Probst

**Affiliations:** Environmental Metagenomics, Research Center One Health Ruhr of the University Alliance Ruhr, Faculty of Chemistry, University of Duisburg-Essen, Essen, Germany; Centre of Water and Environmental Research (ZWU), University of Duisburg-Essen, Essen, Germany; Department of Biodiversity, University of Duisburg-Essen, Essen, Germany; Aquatic Ecosystem Research, University of Duisburg-Essen, Essen, Germany; Aquatic Ecosystem Research, University of Duisburg-Essen, Essen, Germany; Department of Soil and Crop Sciences, Colorado State University, Fort Collins, CO, United States; Environmental Metagenomics, Research Center One Health Ruhr of the University Alliance Ruhr, Faculty of Chemistry, University of Duisburg-Essen, Essen, Germany; Centre of Water and Environmental Research (ZWU), University of Duisburg-Essen, Essen, Germany; Environmental Metagenomics, Research Center One Health Ruhr of the University Alliance Ruhr, Faculty of Chemistry, University of Duisburg-Essen, Essen, Germany; Environmental Metagenomics, Research Center One Health Ruhr of the University Alliance Ruhr, Faculty of Chemistry, University of Duisburg-Essen, Essen, Germany; Centre of Water and Environmental Research (ZWU), University of Duisburg-Essen, Essen, Germany; Centre of Water and Environmental Research (ZWU), University of Duisburg-Essen, Essen, Germany; Aquatic Ecosystem Research, University of Duisburg-Essen, Essen, Germany; Environmental Metagenomics, Research Center One Health Ruhr of the University Alliance Ruhr, Faculty of Chemistry, University of Duisburg-Essen, Essen, Germany; Centre of Water and Environmental Research (ZWU), University of Duisburg-Essen, Essen, Germany; Department of Biodiversity, University of Duisburg-Essen, Essen, Germany; Ruhr University Bochum, Faculty of Biology and Biotechnology, Theoretical and Applied Biodiversity Research, Bochum, Germany; Centre of Water and Environmental Research (ZWU), University of Duisburg-Essen, Essen, Germany; Aquatic Ecosystem Research, University of Duisburg-Essen, Essen, Germany; Centre of Water and Environmental Research (ZWU), University of Duisburg-Essen, Essen, Germany; Department of Engineering and Natural Sciences, Westphalian University of Applied Sciences, Recklinghausen, Germany; Environmental Metagenomics, Research Center One Health Ruhr of the University Alliance Ruhr, Faculty of Chemistry, University of Duisburg-Essen, Essen, Germany; Centre of Water and Environmental Research (ZWU), University of Duisburg-Essen, Essen, Germany; Centre of Medical Biotechnology (ZMB), University of Duisburg-Essen, Essen, Germany

**Keywords:** microbiome, stream, stress, mesocosm, resilience, metagenomics, metatranscriptomics

## Abstract

Rivers face constant anthropogenic stress, resulting in significant changes in microbial community composition. What remains unclear is whether stream microbiomes exhibit distinct resilience patterns in composition and/or activity upon exposure to different stressors. By subjecting 64 river-connected mesocosms to multiple stressors, we show that sediment microbiomes of small lowland rivers are highly sensitive to low flow velocity. This stress results in altered community compositions incapable of mitigating the applied stressor within a two-week timeframe despite functional stability (inferred via metagenomics). Transcriptomics revealed a systematic heat shock response in the community and a highly active, metabolically versatile, uncharacterized anaerobic keystone species. Increases in temperature (+ 3.5°C) or salinity (+ 0.5 mS/cm) elicited minor responses at community and transcriptomic levels (e.g. upregulation of photosystems). Following a two-week recovery, transcriptomic-inferred stress responses vanished completely, underscoring the river microbiome resilience. Given the complex community responses observed at the activity and compositional levels, we conclude that maintaining natural river flow is vital to preventing energy loss and reduced microbiome activity in river sediments.

## Introduction

Despite being hotspots of biodiversity, stream ecosystems make up only 0.58% of Earth’s non-glaciated land surface area [1]. While these niches are species rich, their biodiversity is diminishing at an accelerated pace, jeopardizing global water security [2]. Although several large-scale initiatives, such as the US Clean Water Act, the European Water Framework Directive, the UN’s sustainable development goals for 2030, and the Aichi Targets for 2020 are endorsed, stream ecosystem status has not improved [3, 4]. Even positive developments seen at large scale in Europe for some indicators have come to a halt since 2010 [5]. As such, focusing on freshwater ecosystem conservation was highlighted as a key global task of the new Kunming-Montreal Global Biodiversity Framework endorsed at the 2022 United Nations Biodiversity Conference (COP 15; [6]). This acknowledges the importance of streams in providing essential services for nature and mankind alike, including transport and cycling of carbon and nutrients on a longitudinal scale [7, 8], pollutant removal [9], and potable water [10].

Microorganisms play a crucial role in maintaining river and stream health. Recently, Rodríguez-Ramos *et al.* applied genome-resolved metaproteogenomics to show that complex riverine microbiomes being responsible for carbon and nitrogen cycling are significantly affected by viruses [11]. Moving beyond the borders of stream ecosystems, their microbiomes were deemed a central factor in climate change estimations as rivers were described as one of the “main sources of greenhouse gas (GHG) emission to the atmosphere” [12]. Regarding human needs, diverse microbiomes in various ecosystems can act as “shields” against pathogens [13–15], reduce nutrient load, and remove toxic substances before and during drinking water production from stream water [16, 17]. Despite their importance, the extent to which stream microbiomes change upon exposure to anthropogenic stressors remains poorly understood.

Here, we define a stressor as any environmental variable that, due to anthropogenic interference, exceeds its normal range of variation [18]. The most prominent stressors to stream ecosystems are temperature, nutrient pollution, and hydromorphological changes [19], whose impacts are particularly pronounced in densely urbanized areas [20]. This situation is directly linked to microbial communities since experimental studies have identified direct positive links between their diversity and ecosystem functions (Biodiversity-Ecosystem Functioning (BEF) concept; [21, 22]). Testing the effects of single stressors on microbial communities is straightforward, and can be accomplished via lab experiments [23], field sampling [24], and/or meta-studies [25]. Elucidating the combined effects of multiple stressors, however, is particularly challenging given the number of possible combinations and dependency on parameters and models [26], and is restricted to few studies, e.g. [27, 28]. As such, reports on microbial community responses to multiple stressors and their recovery after stressor release remain scarce. Such knowledge is crucial to identify combinations of stressors that are particularly deleterious to microbial biodiversity and community functions due to synergistic activities and/or interactions [29–31].

*ExStream*, widely used in stream ecology research [29, 32–35], is an experimental system that facilitates analyses of multiple stressors in full-factorial settings with high replication under semi-natural conditions. Nuy *et al.* used the *ExStream* system to evaluate the effects of multiple anthropogenic stressors on leaf litter communities and biofilm tiles [35]. Their results showed that stressors like salinization, fine sediment, and flow velocity elicit distinct response patterns in different microbes. However, they concluded that limited taxonomic resolution and functional information hampered their understanding of the effects of such stressors on complex river microbiomes [35]. Also using the *ExStream* system that was applied in this study, David *et al.* demonstrated that microbes decomposing leaf litter were barely affected by the applied stressors [33]. In another mesocosm study, Romero *et al*. showed that low water levels invoked the greatest bacterial response, while antagonistic effects to temperature and/or pesticides prevailed in the stressor phase [28]. Yet, they conclude that further studies that move from an indoor set up to field conditions are needed. A first-ever synthesis of recovery patterns from disturbed microbiomes showed that aquatic community composition changed significantly compared to pre-disturbance composition and that recovery is environment-specific [36].

Ultimately, responses of microbial communities and their encoded and expressed ecosystem functions to multiple environmental stressors remain understudied. This is particularly true for riverine sediments. Given the essential roles of the riverine sediment microbiomes in maintaining key aquatic ecosystem functions, understanding responses to multiple stressors and their release is of paramount consequence to ecosystem management and restoration. Capitalizing on the BEF concept [21] and the Asymmetric Response Concept (ARC; [37]), a conceptual framework upon which to evaluate the effects of multiple stressors on river biomes, we interrogated river sediment mesocosms as a proxy for the hyporheic zone.

Per Vos *et al.* asymmetric responses can be evaluated by monitoring compositional changes in communities as stress increases and decreases. According to the ARC, individual stress tolerance should be the key determinant in phases of stress, whereas recovery trajectories after stressor release should be shaped by dispersal and biotic interactions [37]. We applied three ubiquitous anthropogenic stressors, including increased temperature and salinity, and low flow velocity, in a full-factorial manner via *ExStream* at the Boye stream in North Rhine-Westphalia, Germany. We investigated sediments during the stressor application phase, and monitored recovery of the microbiome following stressor release. By analysing mesocosms via 16S rRNA gene amplicon sequencing, genome-resolved metagenomics, and metatranscriptomics, we (i) identified compositional and transcriptional community responses, (ii) revealed indicator and keystone organisms under multiple stressor conditions, and (iii) inferred gain or loss of particular ecological functions resulting from stressor exposure. Collectively, these advancements forged an explanatory link between changes in microbiome composition and activity relating to nutrient cycling in rivers. Our results suggest complex interplay of anthropogenic stressors on river microbiome composition and functional response. One key finding is that changes in microbiome composition resulting from low flow velocity cannot compensate for the stressed ecosystem necessitating prokaryotic regulatory mechanisms (e.g. heat-shock responses).

## Materials and methods

### Study site and ExStream system

**Figure 1:**
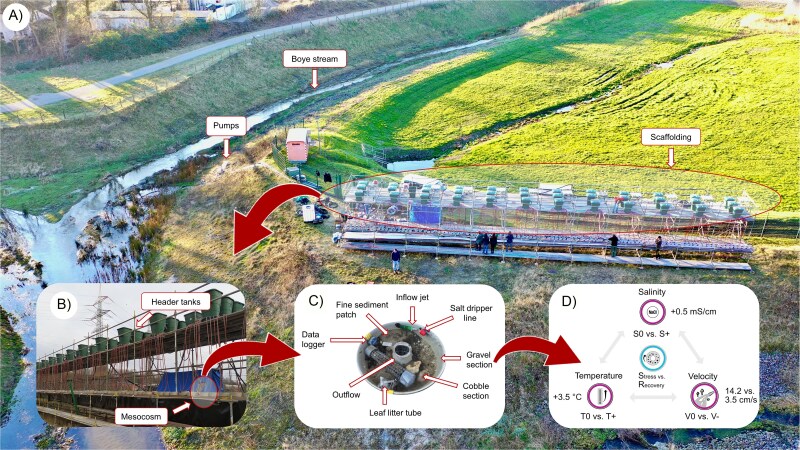
**Experimental design of the highly parallelized full-factorial ExStream mesocosm setup.** (A, B) Stream water was directly pumped from the Boye stream to the scaffolding of three different ExStream setups. An ExStream housing 64 mesocosms was used for this study. (C) Arrangement of the circular mesocosms used in this ExStream experiment. During the stressor phase, low flow velocity was achieved by removing the white cap at the inflow jet. Salinity was added via salt dripper line, and temperature was increased by mixing in-flowing river water with heated river water (constantly measured by data logger). Sediment sampling was performed in the fine sediment patch. (D) Stressor combination applied in full-factorial design with a minimum of three replicates for each phase. Image provided by Philipp M. Rehsen.

The outdoor mesocosm system *ExStream* (Fig. 1) was set up at the Boye stream (51.5533°N, 6.9485°E) and operated from 4 March 2022 to 21 April 2022. The Boye stream, part of the Emscher River system in North Rhine-Westphalia, Germany, has a history of anthropogenic stresses stemming from coal mining, industrial wastewater, and open sewage discharge (early 19^th^ century through 2019; restoration procedures until 2021 [38, 39]). The *ExStream* system [40, 41] is an open mesocosm system in which a continuous supply of stream water flows into multiple circular channels. The setup used in this study has been described previously [33] and is summarized in the Supplementary Information. Briefly, the system housed a two-level scaffold with sediment trap tanks and header tanks on the top level that supplied mesocosms on the bottom level (Fig. 1B). Overflowing water from the sediment trap tanks entered one of four header tanks with 16 connected mesocosms each. All pipes connected to mesocosms had shut-off valves to adjust discharge to 2.1 L/min. Mesocosms had a diameter of 25 cm and a volume of 3.5 L, and were filled with sediment of the stream, gravel, and other substrate to represent the studied stream (see SI).

### Experiment design

Our experiment was divided into three phases: acclimatization, stressor exposure, and recovery. During the 20-day acclimatization phase, all 64 mesocosms were operated *sans* manipulation. During the stressor phase, variations temperature, salinity, and velocity were applied to mesocosms for 14 days in full-factorial fashion (eight replicates per treatment combination; Supplementary File SI1). Temperature was increased by +3.5°C (denoted as “T+” vs. “T0”) compared to ambient stream temperature. Salinity was increased by 0.5 mS/cm (“S+” vs. “S0”) with NaCl via dripper lines, and velocity was lowered from 14.2 to 3.5 cm/s (“V0” vs. “V-”) by removing the in-flow jet. The first endpoint sampling of 32 mesocosms was conducted following the 14-day stressor phase. Four mesocosms per treatment were removed for analysis. The remaining 32 mesocosms continued to run *sans* stressor for 14 days in the recovery phase. As such, each stressor and recovery treatment was represented four replicates. Deviations from planned numbers of replicates and/or offset of stressors due to weather conditions are summarized in the Supplementary Information.

### Sample collection and processing

Sediment cores were collected with a sterile cut 50 ml syringe, procuring the entire depth (~ 5 cm) of the sediment in front of the inflow jet (Fig. 1C). Samples were manually homogenized, subdivided, shock-frozen in liquid nitrogen, and stored at −80°C for further processing. DNA and RNA were extracted from sediments according to methods detailed in the Supplementary Information. Sequencing of metagenomes, metatranscriptomes, and 16S rRNA gene amplicons (cycling conditions for 16S rRNA gene amplification given in Table S3) was performed at CeGat Gmbh (Tübingen, Germany), and raw reads were processed as explained in the Supplementary Information.

### 16S rRNA gene amplicon analyses

Sequenced 16S rRNA gene amplicons were processed with Natrix2 workflow [42], and resulting read counts were normalized via the variance stabilizing transformation (VST) function of the DESeq2 package [43]. Transformed read counts were then converted into PhyloSeq [44] objects with metadata and taxonomic information for further statistical testing and plotting with ggpubr [45], tidyverse [46], and vegan [47]. Amplicon analyses were performed at both the OTU and ASV level.

### Metagenomic assembly and annotation

Quality-checked and filtered metagenomic paired-end reads were assembled, per sample, using MEGAHIT v1.2.9 [48] with the “meta-large” preset. Resulting contigs of at least 1 kbp in length were checked for possible sequencing contaminants using FCS-adaptor [49]. Followingly, open reading frames were predicted using Prodigal (parameters: -m -p meta; [50]) and annotated using DIAMOND blast (DIAMOND version 2.0.15; blastp --fast -e 0.00001 -k 1; [51]) against FunTaxDB (version from August 2022; [52]), based on UniRef100 [53]. Taxonomy was assigned at the contig level based on all proteins detected, as previously described [52]. Mean contig coverage was calculated by mapping quality-checked reads to the assembly using Bowtie2 in sensitive mode [54]. Length and GC content of all contigs were calculated. Assembly statistics are provided in Supplementary File SI2. Pathways encoded in the assemblies were annotated using METABOLIC (v4.0) [55], and stressor effects were evaluated using adonis2 [47] and TOSTER [56], as explained in the Supplementary Information.

### Ribosomal protein S3 marker gene analyses

Based on a phylosift Hidden Markov Model (HMM) set (i.e. DNGNGWU00028; date 20.01.2022) [57] and annotation results from FunTaxDB (see described above) *rpS3* gene sequences were identified in metagenome assemblies [hmmsearch (v3.2), e-value-cutoff of 1E-28]. Clustering of *rpS3* genes (99% nucleotide identity) and coverage estimation was performed as described in the Supplementary Information. To test the significance of stressor impacts on microbial communities, coverage values were rarefied with 100 iterations and a permutational multivariate analysis of variance (PERMANOVA) test (adonis2) was performed based on Bray–Curtis dissimilarity with 999 iterations (R packages included vegan [47], permute [58], and GuniFrac [59]). To infer responsive lineages, each representative sequence was considered (as described in Supplementary Information).

### Binning of metagenomes and incorporation of MAGs from other river ecosystems

Contigs were binned into metagenome-assembled genomes (MAGs) as previously described [60]. In brief, MetaBAT2 [61], ABAWACA [62], and MaxBin2 [63] were used as automatized tools and steps to achieve a manually curated and optimized bin set are described in the Supplementary Information. MAGs from the external database, GROWdb [64, 65] (Supplementary File SI3) were included to minimize the likelihood of overlooking MAGs due to assembly or binning shortcomings. MAGs generated in this study and from GROWdb were dereplicated using dRep (v3.4.3; completeness 75%; contamination 10%; identity of secondary clusters of 95%; [66]), and coverage for dereplicated MAGs was calculated using inStrain (v1.7.6.; “quick-profile”; [67]) with Bowtie2 mappings as input.

MAGs were deemed as present when the breadth of coverage exceeded 0.5. Coverage data for MAGs was used to identify responsiveness to stressor effects during the stressor phase, as described for *rpS3* gene sequences above. Annotation of MAGs was accomplished using GTDB-Tk (v2.1.0 r207; [68]), DRAM (v1.4.6; [69]), and MicroScope [70], as explained in the Supplementary Information.

### Metatranscriptomic analyses

Messenger RNA sequences were filtered from total RNA and mapped to genes from dereplicated MAGs and clustered genes. For the latter, contigs of prokaryotic origin were identified in metagenomic assemblies using EukRep (v0.6.6; [71]) and genes were predicted using Prodigal in meta mode. Resulting genes were clustered at 90% and 95% sequence identity using MMseqs2 (v1806c0c8f7aa4365f9f72c8ea51e947d1e93ccd9, lineclust) [72], which facilitated testing for robustness of the analysis at two different cutoffs (results of the analysis based on 95% are shown in Supplementary Fig. S1 and Supplementary File SI4). Reads were mapped to representative gene sequences in the form of open reading frames (ORFs) using Bowtie2 (−-sensitive; [54]), and counts were calculated using CoverM [73]. Differential expression analyses were performed in R with the DESeq2 package (smallestGroupSize = 3, minimum five channels over smallestGroupSize, FDR/alpha = 0.05; [43]). Shrunken log2 fold changes were calculated with type “apeglm” [74]. Differentially expressed genes [alpha <0.05 and abs(log2FoldChange) > 1] were binned into functional groups manually (Supplementary File SI5).

## Results

A full-factorial mesocosm approach was implemented to evaluate the individual and combined effects of three distinct anthropogenic stressors (i.e. temperature increase [+ 3.5°], salinity increase [+0.5 mS/cm], and low flow velocity [3.5 vs. 14.2 cm/s; Fig. 1D]) on river microbiomes (Fig. 1A-C). Following an acclimation phase of 20 days, stressors were applied to mesocosms for 14 days, followed by recovery *sans* stressors for 14 days. Samples for 16S rRNA gene amplicon, metagenomic, and metatranscriptomic analyses were collected following stressor and recovery phases.

### Low flow velocity is a significant driver of sediment microbiome composition, eliciting greater impacts than either temperature or salinity increase

To elucidate changes in both micro- and macro-diversity to microbial communities upon stressor exposure, we implemented an OTU- and ASV-resolved approach for 16S rRNA genes. Since we are aware of the fact that river ecosystem analyses based on 16S rRNA gene analyses [60] are heavily distorted due to primer and amplification biases, we also inferred microbial community changes by evaluating assembled *rpS3* gene sequences and their relative abundances from metagenomes. Here, sequences were clustered at high sequence similarity (99%), since microdiversity analyses of 16S rRNA genes (based on ASVs) were proven to also be more sensitive to recovery effects (see below and Table 1). Multivariate statistics based on dissimilarity matrices of microbial communities in stressor vs. recovery phases revealed a significant shift in community composition resulting from low flow velocity (adonis2; *P* = 0.001, Table 1). Low flow velocity also yielded in the highest chance-corrected within-group agreement (i.e. greatest difference between community compositions) among all tested stressors via metagenomic data (MRPP; A = 0.008; *P* = 0.001, Table S1). In addition, combinations of stressors (e.g. temperature combined with salinity and low flow) invoked different community effects which were not significant contrary to certain stressors alone (e.g. low flow).

**Table 1:** Statistical testing of stressor impact on microbial community composition based on relative abundance of OTU-resolved and ASV-resolved 16S rRNA gene amplicon data and representative rpS3 gene sequences from metagenomic sequencing.

Descriptor	Stress (n = 32)									Recovery (n = 32)					
	*rpS3*			*16S rRNA ASVs*			*16S rRNA OTUs*			*rpS3*			*16S rRNA ASVs*		
	*R^2^*	*F*	*Pr(>F)*	*R^2^*	*F*	*Pr(>F)*	*R^2^*	*F*	*Pr(>F)*	*R^2^*	*F*	*Pr(>F)*	*R^2^*	*F*	*Pr(>F)*
Temperature	0.04	1.19	0.040	0.02	0.91	0.709	0.02	0.88	0.701	0.03	0.91	0.911	0.02	0.93	0.66
Salinity	0.03	1.04	0.244	0.03	1.04	0.282	0.03	1.13	0.211	0.03	1.04	0.287	0.03	1.03	0.33
Velocity	0.05	1.53	0.001	0.05	1.65	0.001	0.06	2.01	0.0017	0.04	1.12	0.066	0.04	1.27	0.03
Temperature: salinity	0.03	1.01	0.379	0.02	0.93	0.648	0.02	0.87	0.711	0.03	0.97	0.580	0.02	1.03	0.35
Temperature:velocity	0.03	0.99	0.491	0.03	0.98	0.418	0.03	0.97	0.468	0.03	0.91	0.905	0.03	0.86	0.88
Salinity:velocity	0.03	1.01	0.379	0.03	1.06	0.236	0.03	1.12	0.218	0.03	1.06	0.199	0.03	1.15	0.12
Temperature: salinity:velocity	0.03	1.00	0.24	0.02	0.89	0.776	0.02	0.86	0.728	0.03	1.09	0.125	0.03	1.21	0.13

Adonis2 was run with 999 permutations and a model including all single factors and interaction terms based on the marginal effects of the terms as test design. DF = 1 for descriptors. Results of OTU-resolved 16S rRNA gene analyses of the recovery phase and MRPP analysis of rpS3 gene data are presented in Table S1.

Prokaryotic communities subjected to low flow velocity clustered together when visualized with non-metric multidimensional scaling (NMDS) based on 16S rRNA gene relative abundance data (Fig. S2). This was accompanied by a uniform increase in diversity compared to all other perturbations, including both controls and other stressors (Shannon: *P* = 0.0014, Simpson: *P* = 0.015; Fig. S3). However, based on *rpS3* gene sequences, the greatest diversity was observed in treatments exposed to all stressors combined (Fig. S4). While the effect of low flow velocity was consistent across analytical methods (i.e. *rpS3* gene sequence analysis, OTU- and ASV-resolved 16S rRNA gene analyses), different effects were detected for other stressors in the stressor and recovery phases. For instance, *rpS3* gene analysis deemed temperature increase as an additional stressor eliciting a significant microbiome change (adonis2; *P* = 0.040 in *rpS3* gene data versus, *P* = 0.701 and *P* = 0.709 for 16S rRNA gene OTUs and ASVs, respectively [Table 1]). Microbiome analyses based on *rpS3* gene sequences extracted from de novo assembled metagenomes circumvent shortcomings relating to amplification-dependent approaches by being PCR-free, encompassing eukaryotic gene signatures, and enveloping a greater diversity of uncultured prokaryotic microorganisms [75].

**Figure 2:**
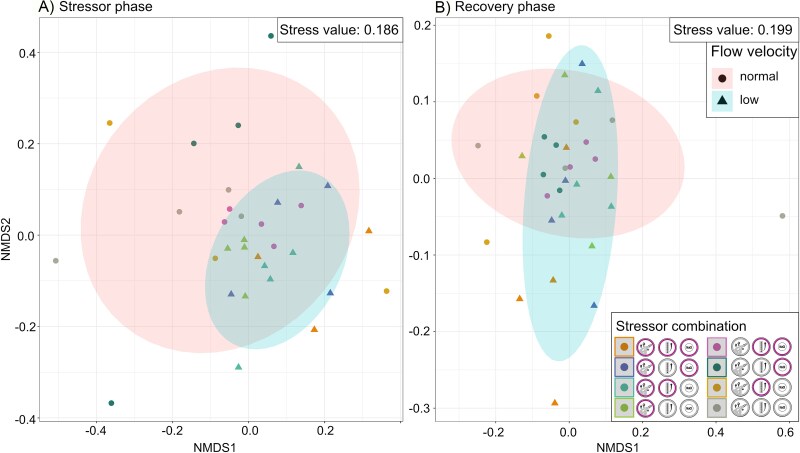
**Low flow velocity causes unifying shift of stream microbiome composition, but the microbiome can recover.** Non-metric multidimensional scaling (NMDS) of normalized abundances of representative rpS3 gene sequences from metagenomic sequencing [Bray-Curtis dissimilarity matrix after rarefaction (100 iterations)] separated for stressor (n = 32) and recovery phase (n = 32). Stressor combinations were grouped by velocity treatment (ggplot2, ellipse level=0.75), indicating a unifying effect of low flow velocity to the stressed microbiome. To underscore that low flow velocity treatments clustered more closely in the stressor than the recovery phase, the ratio of the ellipse size between low flow velocity treatments vs. normal flow velocity treatments was calculated for the two phases. Following stressor exposure, the ellipse enveloping low flow velocity was 31.4% of the size of that enveloping normal flow velocity. This ratio changed to 77.5 % in the recovery phase, reflecting the unifying shift in microbiome composition resulting from low flow velocity.

Only 16S rRNA gene sequences analysis based on ASV relative abundances revealed a significant effect of low flow velocity following the recovery phase (*P* = 0.03, Table S1). This result suggests that low flow velocity had a lasting impact on stream microbiomes. This finding was also reflected in results based on marker gene approaches, which were just above the significance threshold (*P* = 0.06 and *P* = 0.066 for 16S rRNA gene OTUs and *rpS3* gene sequences, respectively (Tables 1 and S1). Timely recovery of native microbiome composition following flow velocity stress was well documented by non-significant community diversity statistics (Table 1) and NMDS based on *rpS3* gene data (Fig. 2). While mesocosms subjected to low flow velocity clustered together after the stressor phase (Fig. 2A), this trend dissipated following the recovery phase (Fig. 2B). However, a less pronounced shift compared to the stressor phase can be detected along NMDS axis 2, which corresponds to the significant effect observed via ASV-resolved 16S rRNA gene analysis.

**Figure 3:**
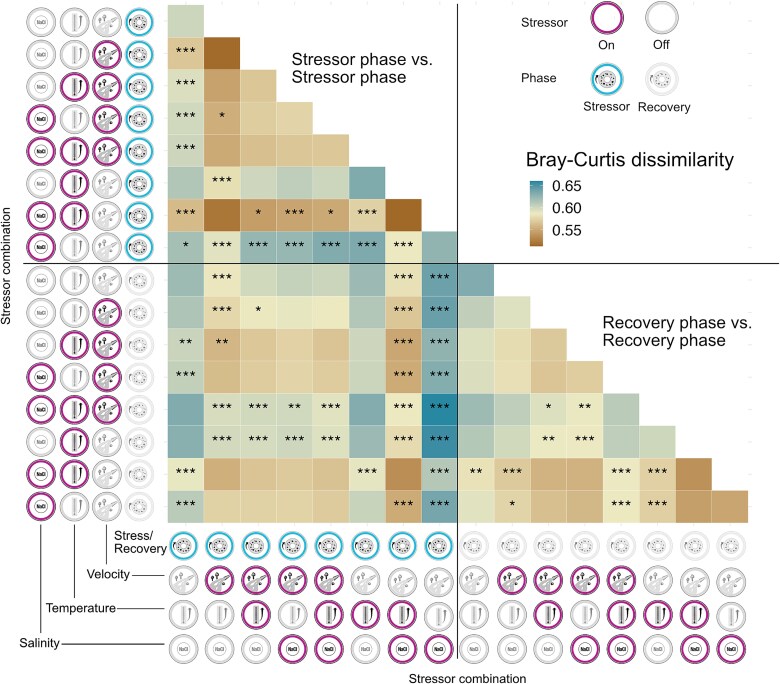
**Treatment-resolved marker gene analysis of stream microbiomes indicates complex community responses to stress and high resilience after stressor removal.** Heatmap displaying pair-wise mean differences in Bray-Curtis distances between all stressor sets (n = 64, all at least in triplicate) based on rarefied relative abundances of representative rpS3 gene sequences. For each comparison, dissimilarity values of respective stressor sets compared to all other stressors were extracted (Wilcoxon test with Bonferroni correction for multiple-testing, asterisks indicate significance level). For example, the control of the stressor phase and channels treated with low flow velocity showed a highly significant difference when the mean of all dissimilarity values of these channels were compared.

To isolate relationships between stressor treatments, the mean pairwise Bray-Curtis dissimilarities between two sets of treatments compared to all other stressor sets was evaluated (Fig. 3). Significant differences in stressed mesocosms were observed. With respect to the recovery phase, only one significant difference was detected when comparing controls to previously stressed mesocosms: combined exposure to increased salinity and temperature. This finding highlights the validity of the mesocosm approach presented to facilitate the monitoring of community development into a new steady state.

All treatments subjected to low flow velocity exhibited comparably low mean dissimilarity to one another, ranging from 0.51 to 0.57 (Fig. 3). As such, analyses of individual treatments (Fig. 3) were in agreement with results of PERMANOVA testing (Table 1) and NMDS (Fig. 2), revealing a uniform shift in microbiome structure owing to low flow velocity. Only the combination of low flow velocity and increased salinity differed from this otherwise universal shift caused by low flow velocity, as a significant difference observed when compared to the treatment with flow velocity solely. By contrast, results for salinity as a sole stressor differed in PERMANOVA testing, which revealed no effect (Table 1), and analyses of individual treatments (Fig. 3)***.*** Increased salinity presented greater dissimilarities and significant differences compared not only to other stressor combinations, but also in its own replicates compared to flow velocity (0.63 for increased salinity vs. 0.51 for low flow velocity).

Upon combining increased salinity with increased temperature, mean dissimilarities, however, dropped and were comparable to stressor combinations with low flow velocity (Fig. 3). While our data suggests that increased salinity has the potential to be a significant stressor, microbial community structure varied too extensively across mesocosms. This variation in response following exposure to increased salinity explains why no significant shifts were detected via PERMANOVA testing (Table 1). Elevated resilience was observed for microbial communities following the recovery phase, as the number of significant differences between treatments (n = 36) decreased from 18 (50%) following the stressor phase to 11 (30%) after the recovery phase.

### Indicator organisms for specific stressors in the river sediments include highly abundant microbes

Sediment microbiomes harbored high biodiversity. This was reflected on the read level in Nonpareil3 analyses with a mean Nonpareil diversity (Nd) value of 23.37 ± 0.11 (n = 64) (Fig. S5), of which sequencing covered between 43.91 and 61.53% (mean of 53.4 ± 3.73%). These values indicate that sequencing accounted for roughly half of the sequence diversity observed in our samples, which is most likely a consequence of the presence of eukaryotic genomes in the samples, which are known to be much more complex than prokaryotic genomes. This shortcoming was overcome by creating an *rpS3* gene sequence database comprising 14 089 lineages based on all 64 metagenomes, which were also used for multivariate analyses (see above).

**Figure 4:**
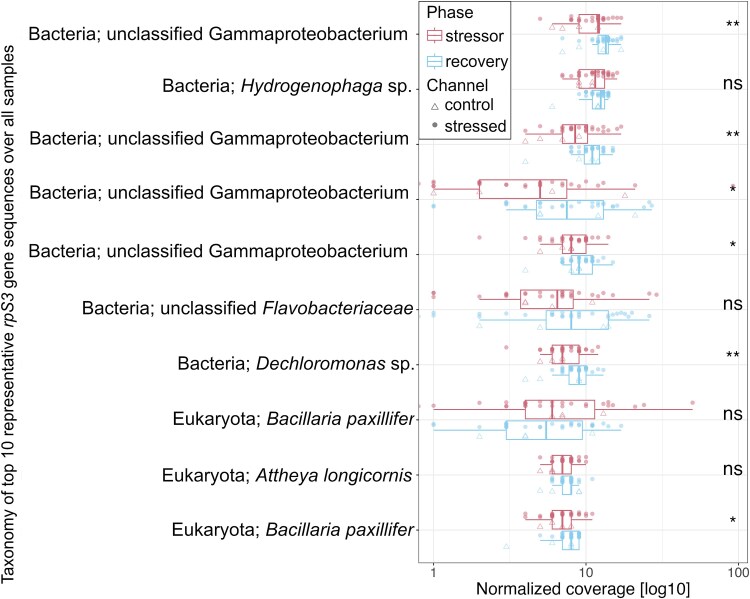
**Microbes which were most abundant across treatments benefit from resilience against applied stressors, even after stressors release.** Box-Whisker plots of the ten most abundant microbes across all samples (ordered from top to bottom based on abundance) with sequencing-depth normalized relative abundances of representative rpS3 gene sequences in stressed and recovered mesocosms. Controls are labeled as triangles. Six of the ten rpS3 gene clusters showed significant increases in relative abundance between stressor and recovery phase (Wilcoxon test). A figure depicting full taxonomy is available as Fig. S6.

Ecosystems dependent on organic carbon turnover are typically driven by highly abundant organisms as they comprise most of the biomass. Consequently, we focused first on the most abundant organisms and changes in their relative abundance during stress response. We investigated these key players based on *rpS3* gene sequence abundances, homing in on differences between stressed and recovered mesocosms (Fig. 4 and Fig. S6). Overall, Betaproteobacteria, Gammaproteobacteria, and eukaryotic Bacillariophyta dominated the microbiomes across all treatments. However, several organisms of identical taxonomic annotation were present amongst the 10 most abundant taxa due to our clustering approach, which was geared towards resolving microdiversity rather than species (clusters based on 99% gene similarity). Six of the 10 most abundant *rpS3* gene clusters showed a significant increase in relative abundance during recovery compared to stressor application confirming the asymmetric nature of river microbiome responses to stressors, ultimately resulting in different microbiome compositions following the recovery phase.

In most instances, the most abundant *rpS3* sequences in control mesocosms were less abundant than they were in treated mesocosms (Fig. 4). We conclude that the most abundant microbial taxa exhibited greater tolerance to the stressors applied than the less abundant, more sensitive taxa. This advantage during the stressor phase seems to carry over and benefit the most abundant taxa via outcompeting sensitive taxa in the recovery phase. This recovery mechanism might be perceived as a process of “depauperate reassembly” as recovery of the microbiome is driven by a limited suite of dominant and tolerant microbes that managed to further increase in number in the post-disturbance environment compared to taxa which were sensitive during stressor phase.

**Figure 5:**
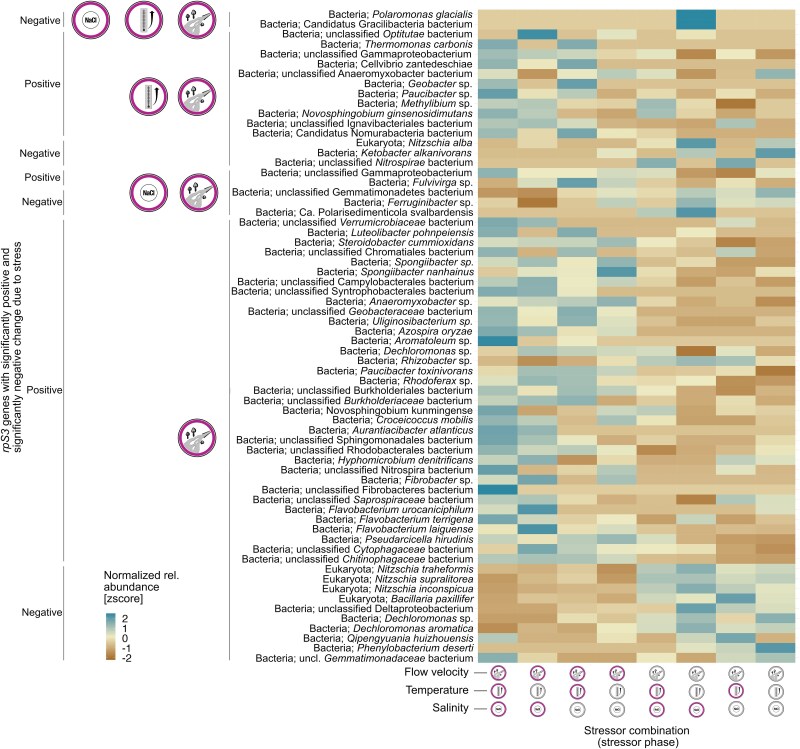
**Metagenomic marker gene analysis sheds light on sensitive microbial taxa that respond to low flow velocity indicating a significant impact on, e.g., diatoms.** Microbial sensitivity to low flow velocity based on sequencing-depth normalized representative rpS3 gene sequence abundances (ANOVA followed by TukeyHSD; adjusted p-value < 0.01). If numerous stressor effects per taxon were significant, only the lowest p-value was presented. For example, the taxon at the very bottom, an unclassified Gammatimonadaceae bacterium, was negatively affected by low flow velocity, although its individual relative abundance was not uniform across stressor sets. For relative abundance changes due to increased salinity or temperature, please see Fig. S7 or S8, respectively. All taxa responding significantly to a stressor are summarized in Supplementary File SI6.

To elucidate microbiome response at taxon level, i.e. changes in *rpS3* gene signature relative abundances resulting from stressor exposure, we identified 110 potential indicator taxa (Tukey’s HSD post-hoc testing; adj. *P*-value <0.01; Fig. 5, Fig. S7, and Fig. S8, Supplementary File SI6). Sixty-six organisms spanning 11 phyla responded either negatively or positively to decreased flow velocity, 21 of responded only when a secondary stressor was applied (Fig. 5).

Important indicator taxa for low flow velocity are diatoms, e.g. *Nitzschia* sp., which responded negatively and for example *Flavobacterium* spp., which responded positively. With respect to increased temperature (Fig. S8), certain thermophilic bacteria like *Schlegelella thermodepolymerans* responded positively as well as two representative sequences annotated as *Hydrogenophaga* spp.. Parasitic organisms like Candidatus Gracillibacteria, CPR SR1 bacteria, and a eukaryotic endosymbiont of *Kryptoperidnium foliaceum* responded negatively to temperature increase suggesting that these organisms or their hosts are outcompeted following a 3.5°C increase in temperature. Increased salinity affected stream microbiomes to a lesser extent compared to the other stressors (Fig. S7), in agreement with multivariate analyses (Table 1). However, *Aurantiacibacter atlanticus*, a lineage of marine proteobacteria, responded positively to increased salinity. Putative thermophiles responding to increased temperature and salt-adapted organisms responding to increased salt link physiological knowledge to changes in gene signatures, thereby confirming our analysis approach. The 110 identified indicator organisms expand our knowledge of microbial indicators for stressed river ecosystems substantially.

### Organism-specific changes in function are related to stressor application and recovery

**Figure 6:**
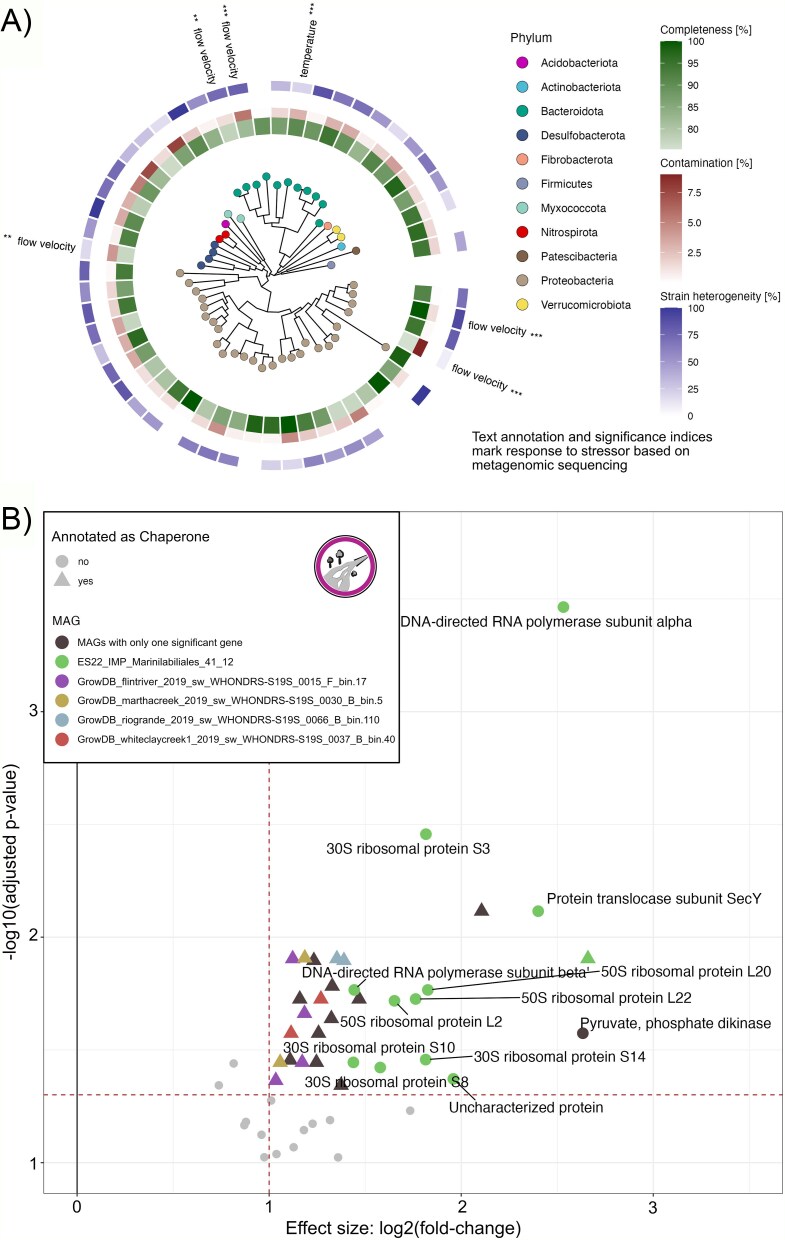
**Upregulation of chaperones is a key response mechanism to low flow velocity detected in recovered MAGs.** Phylogenetic, stressor response, and transcriptomic analysis of MAGs from both ExStream experiment and GrowDB [64]. (A) Dereplicated MAGs (n = 55; completeness > 75%, contamination < 10%) were dominated by Proteobacteria. One MAG was annotated as an Archaeon and is thus not shown in this bacterial tree. When evaluated for differential abundances owing to various applied stressors (n = 32 channels), five MAGs responded positively to low flow velocity and one MAG to increased temperature (text annotation with significance index as asterisks). (B) Metatranscriptomic reads were mapped to the suite of MAGs and evaluated for differential expression due to low flow velocity using DESeq2 [43] (n = 32 for stressor phase; log2[fold-change] > 1 and adjusted p-value < 0.05; all gene annotations in Supplementary File SI10; [43]). Eleven MAGs encoded only one gene responding to low flow velocity. Chaperones were identified as indicator genes for low flow velocity. Differential expression resulting from increased temperature and all stressors combined is shown in Fig. S10. For all other stressor combinations, no differentially expressed genes could be identified following the stressor and recovery phases.

To link changes BEF, genome-resolved metagenomics were coupled to metatranscriptomics approaches. This facilitated identification of differentially expressed genes of MAGs resulting from stressor exposure. A suite of 55 at least medium-quality MAGs according to MIMAG standards was procured ([76]; quality statistics of MAGs, functional annotation from DRAM, and taxonomic classification from GTDB-Tk are provided in Supplementary Files SI7-SI9), most of which were annotated as being Proteobacteria. High strain heterogeneity (47.6% $\pm$ 30.34%) was detected across all MAGs (Fig. 6A), which is similar to many soil microbiomes [77–79] and explains the sparse recovery of MAGs from river sediments.

Upon testing for responses to applied stressors based on read mapping of metagenomic data, five MAGs and one MAG were significantly positively impacted by low flow velocity (Tukey-HSD; max. p.-adj. < 0.01) and increased temperature (Tukey-HSD; p.-adj. < 0.001), respectively. This was consistent with 16S rRNA gene amplicon and *rpS3* gene sequence data (Table 1). The latter, a member of class Bacteroidia, was only present in samples subjected to increased temperature (Fig. S9), rendering this organism an indicator species. This organism possessed six genes that were significantly differentially expressed upon exposure to increased temperature (Fig. S10), two of which encode uncharacterized proteins, three others encode T9SS type A sorting domain-containing proteins, and the last encodes a HU family DNA-binding protein (Supplementary File SI10). The latter two proteins play crucial roles in maintaining cell integrity and metabolic functioning. For instance, T9SS type proteins are known to contribute to virulence [80], motility [81], and S-layer formation [82] in Bacteroidetes, while HU DNA-binding proteins partake in DNA coiling, repair, and transcription [83].

Thirty-three genes present in at least one of the MAGs were significantly upregulated upon exposure to low flow velocity (Fig. 6B). Of these, 21 encoded chaperone-related proteins and seven ribosomal proteins (Supplementary File SI10). These results demonstrate that several organisms responded by upregulating protein production (translation via ribosomes for which ribosomal proteins are necessary) and augmenting their stress response with more chaperones, which facilitate proper protein folding [84, 85].

**Figure 7:**
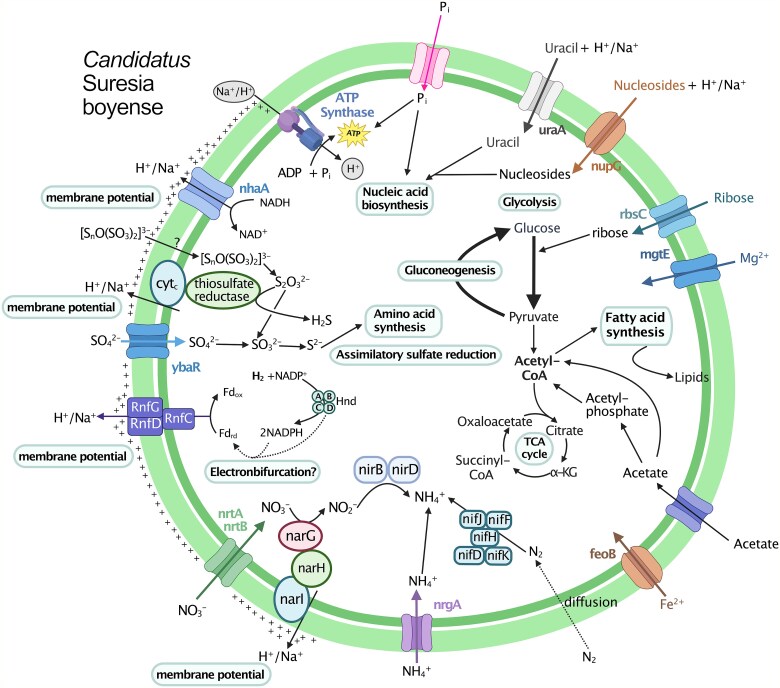
**The metabolically versatile keystone organism named Candidatus “Suresia boyense” exemplifies the hidden diversity of stream microbiomes.** Metabolic capacity of an identified keystone organism in the analyzed microbial community of the Boye: MAG Marinilabiliales_41_12 (GTDB taxonomy; phylum Bacteroidota, family Prolixibacteraceae) named Candidatus “Suresia boyense” after Bernd Sures who pioneered the use of the Boye stream as a model ecosystem for restoration. We used one MAG to manually reconstruct of this metabolic map (n = 1) using the MicroScope platform [70], followed by visualization via BioRender.com.

The MAG bearing the most differentially expressed genes (in short, Marinilabiliales_41_12) belonged to the family *Prolixibacteraceae*, and, according to metatranscriptomic data, expression of its *rpS3* gene was significantly upregulated upon low flow velocity. Metagenomic data, however, did not show upregulation of this gene, and it was only detected in three samples per read mapping (Fig. 5). Taken together with the upregulation of polymerases, this indicates low abundance but high activity of this organism rendering it a typical keystone species [86]. We investigated the encoded metabolism of this keystone organism’s genome (86.82% complete, 1.53% contamination, ~ 4 Mbp) via manual annotation using the MicroScope platform [70], deciphering potential carbon, nitrogen, sulfur and phosphorus sources (Fig. 7). The genome encoded a variety of transporters evident of a heterotrophic lifestyle, which was corroborated by the presence of near-complete and active glycolysis and TCA cycles. The primary sulfur source for amino acids appeared to be assimilatory sulfate reduction, while nitrogen was sourced from either ammonia uptake via transporters or strictly anaerobic nitrogen fixation via NifHD (three subunits encoded alongside one another and next to a nitrogenase iron protein). While membrane-bound nitrate reduction to ammonia via nitrite is fully encoded for energy conservation, we also identified tetrathionate to thiosulfate conversion with the latter potentially being reduced to sulfite (thiosulfate/3-mercaptopyruvate sulfurtransferase potentially contributes its product to assimilatory sulfate reduction) or sulfide (via thiosulfate reductase with a membrane-bound cytochrome subunit). Genes for portions of these sulfur pathways were determined to be active by transcriptomic analyses. Subunits of an encoded NADH oxidoreductase likely energize the membrane via oxidation of NADH. This heterotrophic and likely chemolithotrophic keystone exhibited remarkable metabolic versatility, rich in pathways that allow it to metabolize a wide variety of nutrients and energy sources. Low flow velocity limits limited oxygen supply into sediments, rapidly resulting in microhabitats that favor this strictly anaerobic microorganism.

### While encoded ecosystem functions remain stable at larger scales, they are rather sensitive at the gene expression level

Since genome-resolved metagenomic techniques fail to capture the complete functional capacity of a given microbiome, we also analysed metagenomic and metatranscriptomic data at the gene level. No significant changes resulting from stressor exposure could be identified using multivariate statistics based on normalized relative abundances of metabolic modules present in metabolic assemblies, as detected by METABOLIC [55] (Table S2), which included carbon and sulfur cycling, nitrogen turnover, and oxygen metabolism (see Supplementary File SI11). As the absence of significant differences does not imply significant similarity, relative abundances of encoded functions were tested for equivalence using the TOSTER package [56] (Fig. S11). This analysis revealed that the extent of stressors applied directly impacted the number of equally shared functions detected. More specifically, when all stressors were applied, no functions were significantly equivalently encoded compared to the control or channels subjected to one stressor. When temperature was increased as a sole stressor, functions were only equally shared with channels that also experienced increased temperature as a stressor. Given the absence of significant differences in prokaryotic functions and the frequent detection of their similarity, we conclude that they remained mostly stable across all stressor tests. However, changes in eukaryotic communities, (e.g. decreased number of diatoms upon low flow velocity) imply that at least system-level production was substantially affected.

**Figure 8:**
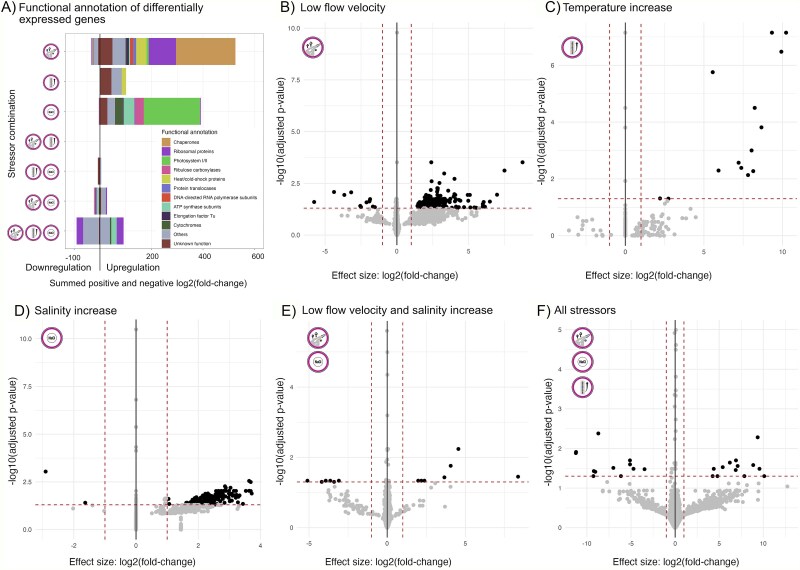
**Gene-level functional metatranscriptomic analysis reports distinct response mechanisms to applied stressors, especially to low flow velocity and increased salinity.** (A) Overview of differentially expressed genes and their functional annotation following stressor application. Up- and downregulated genes were summarized and manually placed into functional groups, revealing that low flow velocity and increased salinity lead to specific responses while other stressors elicit nonspecific response, if any at all. (B-F) Metatranscriptomic reads were mapped to all prokaryotic genes clustered at 90% similarity and tested for significance (dashed lines represent log2[fold-change] > 1 and adjusted p-value < 0.05). Plots depict treatments following the stressor phase; only treatments yielding more than two differentially expressed genes are shown. Low flow velocity results in drastic upregulation of prokaryotic genes (194; 41.8% annotated as chaperones), while increased salinity triggers upregulation of plastid photosystems. There were only two genes significantly differentially regulated following the recovery phase. All annotations for significant genes are given in Supplementary File SI5.

To evaluate the robustness of the analyses, the effects of stressors on gene expression was analysed applying gene clustering thresholds of 90% (Fig. 8) and 95% (Fig. S1). Since no major differences were observed between the two thresholds, only results of the analysis with a 90% cutoff are discussed further, representing a more functional-focused approach than the species-level cutoff of 95%. Focusing on expression patterns of genes of prokaryotic origin [71] across all treatments (Fig. 8a-f), we observed the salinity-driven upregulation of 164 genes, 94 of which were annotated as belonging to a “photosystem” of plastids (see annotation in Supplementary File SI5). Gene expression was most affected by low flow velocity, resulting in twelve downregulated and 182 upregulated genes, 41.8% of which were annotated as “chaperone”. Twelve upregulated genes were related to heat-shock proteins (“hsp20”, “Small heat shock protein”, and “heat shock protein”). Only 54 genes were affected by other stressors, including increased temperature (14), low flow velocity AND increased salinity (13), salinity increase AND increased temperature (2), or all stressors combined (25).

Comparing response patterns to stressor combinations based on functional groups (Fig. 8A), low flow velocity activated a general microbial stress while increased salinity led to a more distinct response. Other stressors, either alone or in combination, did not trigger specific functional signals, if any at all. As such, specific responses resulting from low flow velocity and increased salinity were undetectable when combined with other stressors. Since only two genes were detected as differentially expressed genes following the recovery phase, we conclude that gene expression-dependent stress responses persist for but a short period of time after the stressor is removed.

## Discussion

Stream ecosystems are under considerable anthropogenic stress, on a global scale [87, 88]. Efforts to restore stressed streams often fall short of complete recovery. This is, in large part, due to a lack in understanding how previous stressor exposure shapes recovery trajectories following stressor release [37]. Building upon the BEF concept [22] and our previously published asymmetric response concept (ARC) [37], we evaluated these theoretical frameworks on river sediment microbiomes. The ARC suggests that the primary mechanisms of stream community assembly, i.e. stress tolerance, dispersal, and biotic interactions, play roles to varying extents during stressor increase and after stressor release. We show that integration of stress tolerance of microbiomes is key for predicting their response to, e.g. low flow velocity. Evaluations of the most abundant taxa across stressors suggests that stress tolerance during stressor exposure provided benefits after stressors were removed with a headstart compared to more sensitive taxa considering relative abundance measures indicating a form of “community closure” [89].

Low flow velocity caused canalization (reduced variability) while increased salinity as a stressor enhanced noise (increased variability) in the microbiome. However, even the latter became canalized when increased temperature was applied as a secondary stressor [90]. Responses to increased salinity were only detectable at taxa and gene expression levels without the uniform response in community change (Adonis test) as observed for low velocity and increased temperature. This might be explained by the history of this model stream, having been previously impacted by mining and used as an open sewer system, likely selecting for a microbiome adapted to greater salt concentrations [39, 91].

Similar findings have been reported for eukaryotic communities, microbial decomposers, and ecosystem functions [33, 40]. This would suggest long-term adaptation of river microbial communities, which should be evaluated in future endeavors. While the stream studied is somewhat special in history and location, data amassed in 2021 show that only 37% of EU surface waters were in good or high ecological status rendering the Boye one of many stressed rivers in Europe [92]. While this study showed a high resolution at the taxonomic level and identified relevant indicator species and activity changes in microbial metabolism, countless *ExStream* setups that include pristine streams need to be set up across the continent to determine whether the results discussed here are generalizable or applicable only to streams that have undergone considerable anthropogenic stress in the past. It is plausible to suggest that historically degraded streams bear greater stress tolerance due to epigenetic imprinting compared to pristine streams. In turn, the rich biodiversity of pristine streams should coincide with elevated resilience to stressors.

Throughout this full-factorial outdoor mesocosm study, bacterial river communities from sediments responded significantly to low flow velocity, not only via compositional changes in community structure but also by upregulating chaperones proteins. This may be a clear sign of an energy-demanding stress response [93, 94]. Such processes can rapidly drain cellular energy [95, 96], hinder microbiomes’ fitness, and reduce its capacity for nutrient turnover in rivers—ultimately culminating in gross biofouling and stagnation. For instance, it has been shown that key water quality metrics like the biochemical oxygen demand are substantially influenced by changes in flow velocity, e.g. as a result of run-of-river dams [97]. Another energy-draining process is the upregulation of heat-shock proteins, which occurred upon exposure to low flow velocity and increased temperature. Such upregulation increases bacterial resistance to physical and chemical stressors [98, 99]. The upregulation of photosystem encoding genes resulting from increased salinity, also observed along a river-to-sea continuum [100], suggests that more free energy is required to mitigate the stress currently confronting the microbiome. The metabolic versatility and activity of the novel keystone MAG, *Suresia boyense,* appear to help this organism thrive under stressed conditions.

Taken together, adaptations to the three stressors applied have important implications for primary production in stream beds, not only due to shifts in gene expression, but also changes in relative abundance, e.g. a lower relative abundance of diatoms resulting from low flow velocity. Given that rivers and streams factor largely carbon dioxide burial from terrestrial inputs (0.3 Pg C yr^−1^; [12]) and diatoms account for roughly 20% of the planet’s primary production [101, 102], we posit that low flow velocity of rivers may negatively impacts primary production. As primary production is exceedingly important to climate change (river gross primary production 0.07 Pg C yr^−1^; [12]), ensuring that rivers maintain natural flow variations, with both fast and slow flowing sections, might markedly improve provisions of essential ecosystem functions [9, 103] and recovery from stressed phases. Nutrient cycling might become even more relevant in particular seasons (e.g. leaf litter decomposition in autumn [100]). To address seasonal effects, follow-on mesocosm experiments must be conducted that either run continuously year-round or commence at different times of the year.

In summary, our results clearly demonstrate that low flow velocity (and to a lesser extent, increased temperature) significantly alter stream sediment microbiomes at community and organism levels. While there is redundancy in encoded functions, the microbiome was forced to acclimatize, at least in the short-term, via transcriptomic stress responses. We did not detect a fully functional microbial community *sans* stress response. As such, exceedingly rich bacterial diversity available via dispersal did not establish new dominant taxa capable of mitigating the experimental conditions as a stress-free environment in the tested time frame of 2 weeks. Consequently, the restructured microbiome resulting from 2 weeks of exposure to low flow velocity was not yet adapted to the applied stress conditions, and as such, needed to upregulate its heat stress response. We conclude that such stress responses represent substantial energy sinks, diverting energy from other critically important ecosystem processes (e.g. nitrogen turnover) and rendering the entire microbiome less productive. However, these changes were reverted following two weeks of recovery, underscoring the resilience of the river sediment microbiomes. Ultimately, we unveiled a key prerequisite for restoration initiatives: assurance that rivers and streams are allowed to flow freely, at natural unimpeded velocities, such that the health of the stream sediment microbiome is maintained and communities can perform their respective ecosystem services efficiently.

## Supplementary Material

Supplementary_material_ycaf079

## Data Availability

All raw sequencing data, 16S rRNA gene data, and MAG sequences used in this study have been deposited at SRA or GenBank, and are accessible under the BioProject PRJNA1085635. The MAGs from GROWdb are accessible at KBase [64].

## Code availability

Code for statistical analysis and figure creation has been deposited at the ProbstLab GitHub page (https://github.com/ProbstLab/publication-associated_scripts).
