# Supplementary material for: Complex compositional and metabolic response of river sediment microbiomes to multiple anthropogenic stressors
Source: ISME Commun. 2025 May 17;6(1):ycaf079. doi: 10.1093/ismeco/ycaf079 (PMC13245187; doi:10.1093/ismeco/ycaf079)
Supplement: Supplementary_material_ycaf079 [file supplementary_material_ycaf079.zip › Stach_Deep_2025_Supplementary_Information.pdf]

Supplementary Information for

Complex compositional and metabolic response of river sediment microbiomes to multiple anthropogenic stressors

Tom L. Stach[1,2,✢], Aman Deep[3,✢], Iris Madge Pimentel[4], Dominik Buchner[4], Mikayla A. Borton[5], André Soares[1,2], Jörn Starke[1], Till L.V. Bornemann[1,2], Philipp M. Rehsen[2,4], Ken L. Dreger[1], Jens Boenigk[2,3], Matthijs Vos[6], Florian Leese[2,4], Daniela Beisser[2,7]* and Alexander J. Probst[1,2,8,*]

[1]Environmental Metagenomics, Research Center One Health Ruhr of the University Alliance Ruhr, Faculty of Chemistry, University of Duisburg-Essen, Essen, Germany
[2]Centre of Water and Environmental Research (ZWU), University of Duisburg-Essen, Essen, Germany
[3]Department of Biodiversity, University of Duisburg-Essen, Essen, Germany
[4]Aquatic Ecosystem Research, University of Duisburg-Essen, Essen, Germany
[5]Department of Soil and Crop Sciences, Colorado State University, Fort Collins, Colorado, USA
[6]Ruhr University Bochum, Faculty of Biology and Biotechnology, Theoretical and Applied Biodiversity Research, Bochum, Germany
[7]Department of Engineering and Natural Sciences, Westphalian University of Applied Sciences, Recklinghausen, Germany
[8]Centre of Medical Biotechnology (ZMB), University of Duisburg-Essen, Essen, Germany

✢ These authors contributed equally to this work.

* Address correspondence to:
alexander.probst@uni-due.de and daniela.beisser@w-hs.de

Table of contents

1. Supplementary results 3
   a. Gene-level functional metatranscriptomic analysis based on clustering at 95% similarity 3
   b. Prokaryotic community analysis based on 16S rRNA gene amplicon sequencing data. 4
   c. Significance testing of rpS3 gene and OTU-resolved 16S rRNA gene data. 5
   d. Alpha diversity of 16S rRNA gene amplicon sequencing data and rpS3 gene sequences. 6
   e. Nonpareil curves of metagenomic samples. 7
   f. Most abundant microbes across all samples with full taxonomy information. 8
   g. Species-specific stressor response based on rpS3 gene analysis. 9
   h. Metatranscriptomic analysis of MAGs. 12
   i. Statistical testing of encoded ecosystem functions. 12
2. Supplementary materials and methods 15
   a. Experiment design and mesocosm setup. 15
   b. Contamination assessment of ExStream mesocosm system. 15
   c. Temperature as stressor. 15
   d. Salinity as stressor. 16
   e. Velocity as stressor. 16
   f. Deviation from the number of replicates and offset of stressors. 16
   g. DNA and RNA extraction and sequencing. 16
   h. Transcriptomic RNA sequencing and read processing. 17
   i. 16S rRNA gene amplicon analysis. 17
   j. Metagenomic sequencing and quality control. 18
   k. Ribosomal protein S3 marker gene analysis. 19
   l. Binning of assembled metagenomes into MAGs and annotation. 19
   j. Functional annotation and equivalence testing. 20
3. References 21

1. Supplementary results

a. Gene-level functional metatranscriptomic analysis based on clustering at 95% similarity

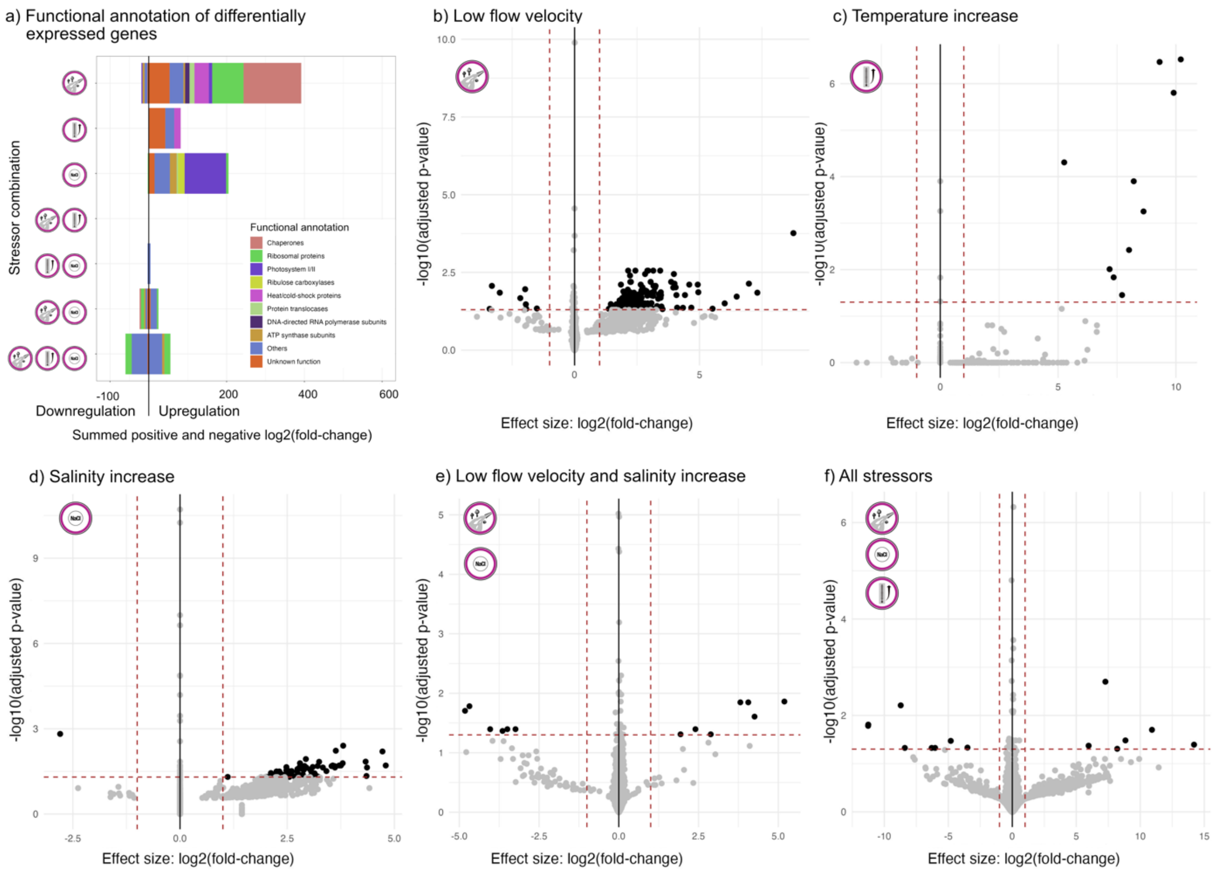

Figure S1: Functional metatranscriptomic data based on gene clustering at 95%. a) Overview of differentially expressed genes and their functional annotation after stressor application. Up- and downregulated genes were summed up respectively and manually grouped into functional groups revealing that low flow velocity and salinity increase lead to specific responses while other stressors are rather nonspecific if having a response at all. b-f) Metatranscriptomic reads were mapped to all prokaryotic genes clustered at 95% similarity and tested for significance (dashed lines represent log2(fold-change) > 1 and adjusted p-value < 0.05). Plots refer to the treatments after the stressor phase and all annotations for significant genes are given in SI4.

b. Prokaryotic community analysis based on 16S rRNA gene amplicon sequencing data.

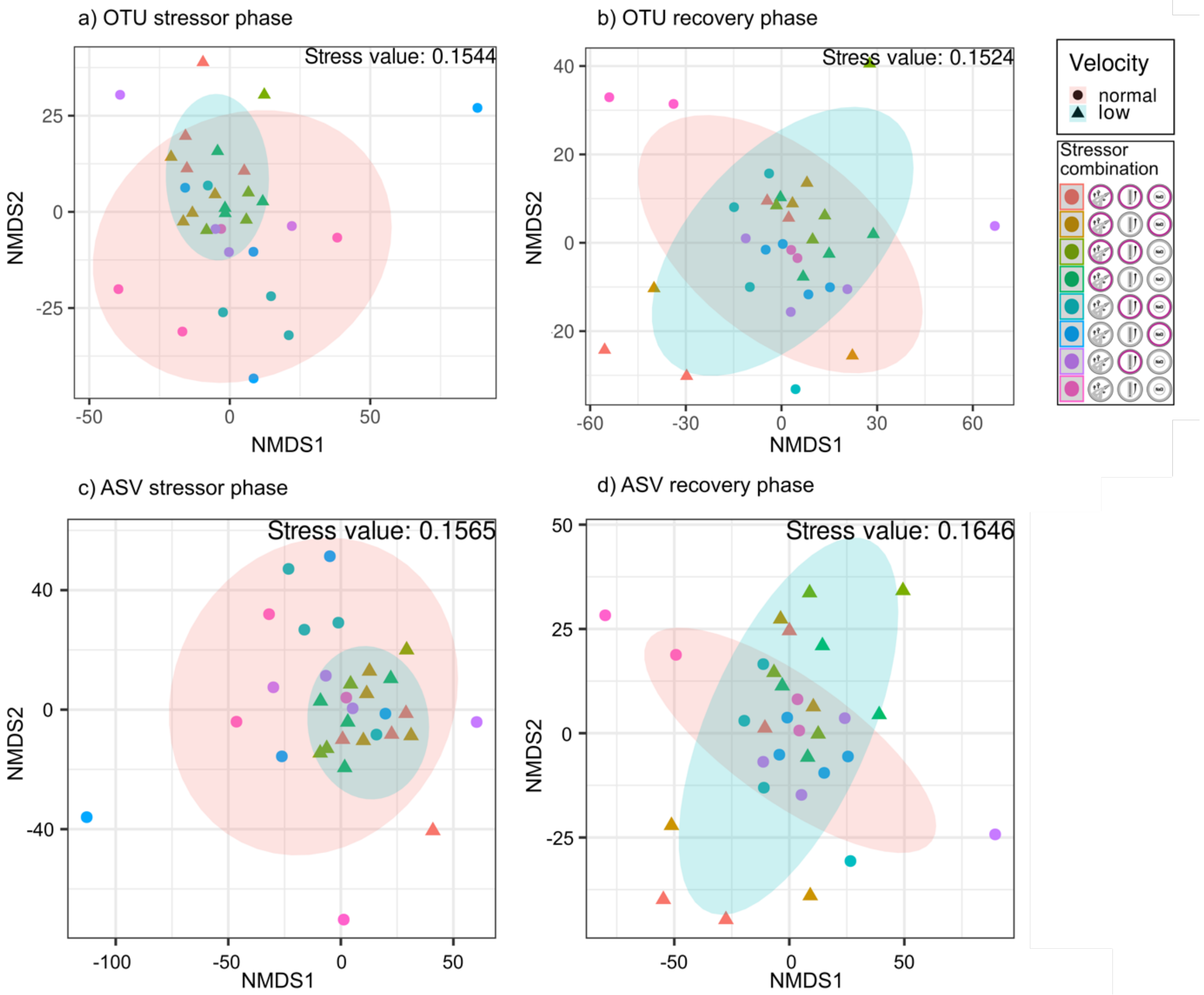

Figure S2: Non-metric multidimensional scaling (NMDS) of OTUs (a and b) and ASVs (c and d) from 16S rRNA amplicon sequencing (Bray-Curtis dissimilarity matrix) separated for stressor (n=32) and recovery phase (n=32), respectively. Stressor combinations were grouped by velocity treatment (ggplot2, ellipse level=0.75) indicating a unifying effect of low flow velocity to the stressed microbiome.

c. Significance testing of rpS3 gene and OTU-resolved 16S rRNA gene data.

Table S1: Statistical testing of stressor impact on microbial community structure based on relative abundance of OTU-resolved 16S rRNA gene amplicon data and representative rpS3 gene sequences from metagenomic sequencing. This table is a supplementary information for Table 1 of the main manuscript. Adonis2 was run with 999 permutations and a model including all single factors and interaction terms based on the marginal effects of the terms as test design. MRPP was grouped for descriptors individually and ran with 999 permutations. DF=1 for descriptors.

Descriptor	16S rRNA OTUs – adonis2			rpS3 gene - MRPP					
	Stress (n=32)			*Stress (n=32)*			*Recovery (n=32)*		
	R^2	*F*	*Pr(>F)*	*A*	*δ*	*p-value*	*A*	*δ*	*p-value*
velocity	0.04	1.33	0.06	0.008	0.58	0.001	0.002	0.54	0.076
salinity	0.03	1.08	0.277	0.00008	0.58	0.353	0.00008	0.54	0.408
temperature	0.02	0.81	0.82	0.003	0.58	0.032	-0.001	0.54	0.893
velocity: salinity	0.03	1.21	0.150						
velocity: temperature	0.02	0.83	0.781						
salinity: temperature	0.03	1.11	0.246						
velocity:salinity: temperature	0.03	1.20	0.134						

d. Alpha diversity of 16S rRNA gene amplicon sequencing data and *rpS3* gene sequences.

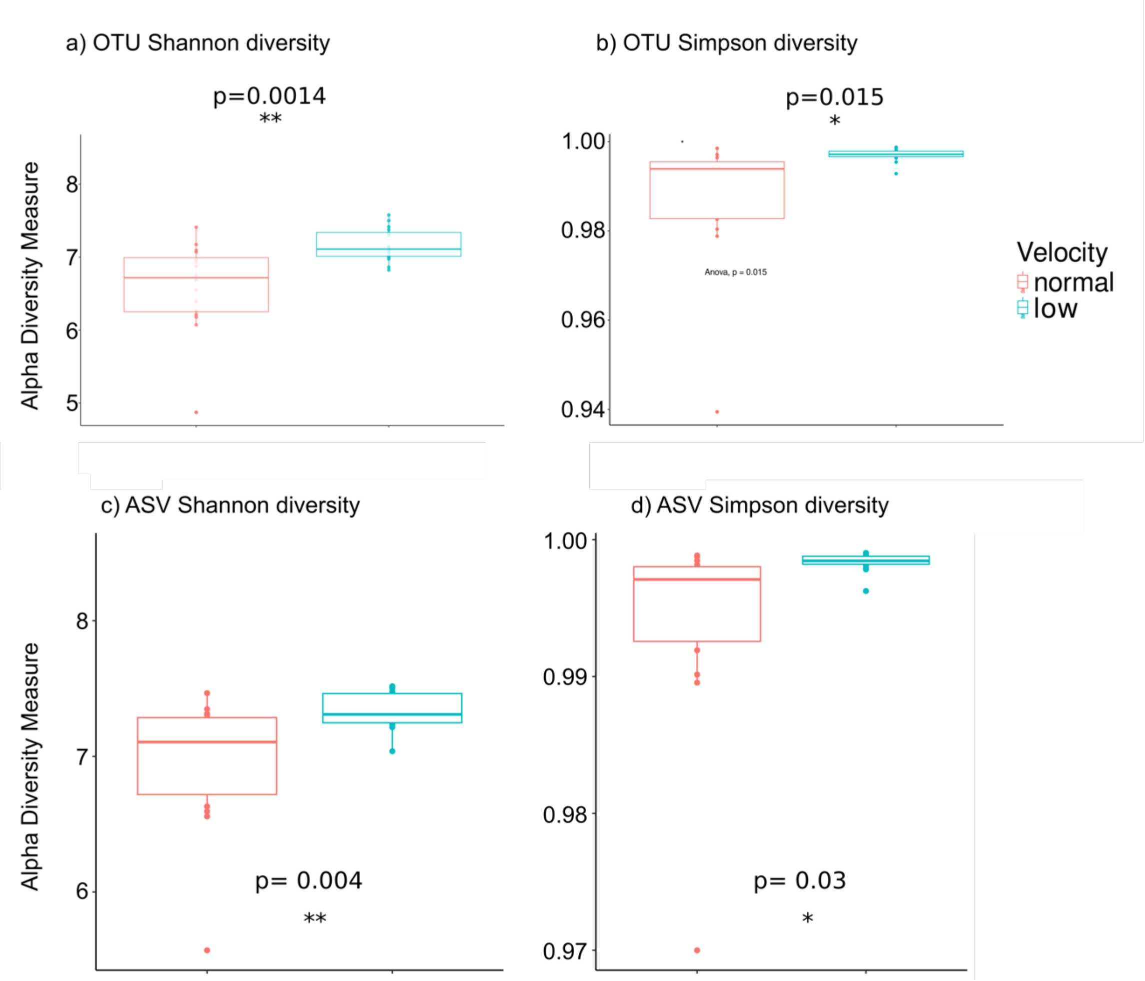

Figure S3: Diversity indices based on bacterial community (16S rRNA amplicon) under low and normal velocity during the stressor phase based on OTUs (a and b) and ASVs (c and d), respectively. Alpha diversity plot showcases the within-sample diversity of bacterial communities. The x-axis corresponds to samples from reduced and normal velocities, while the y-axis captures alpha diversity indices, offering insights into species richness and evenness. The significance of velocity response was analyzed with an ANOVA test from the stat_mean_compare function of the R package ggpubr [1].

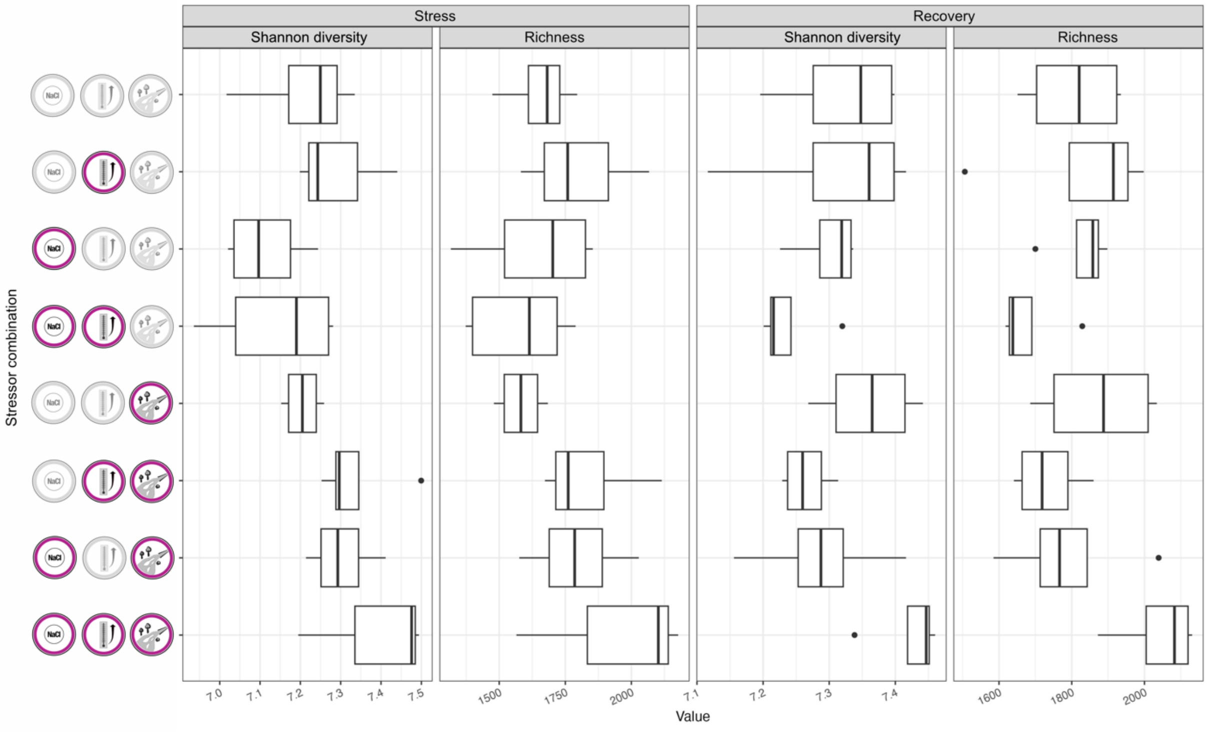

<u>Figure S4:</u> Shannon diversity and Richness of microbial community based on *rpS3* gene sequences. Samples were separated according to the stressor combination. In the stressor phase, flow velocity results in samples with more similar alpha diversity metrices compared to the other treatments. Generally, differences based on alpha diversity are low.

<u>e. Nonpareil curves of metagenomic samples.</u>

The diversity of the microbial community and its covered fraction by metagenomic sequencing was estimated using Nonpareil3 [2].

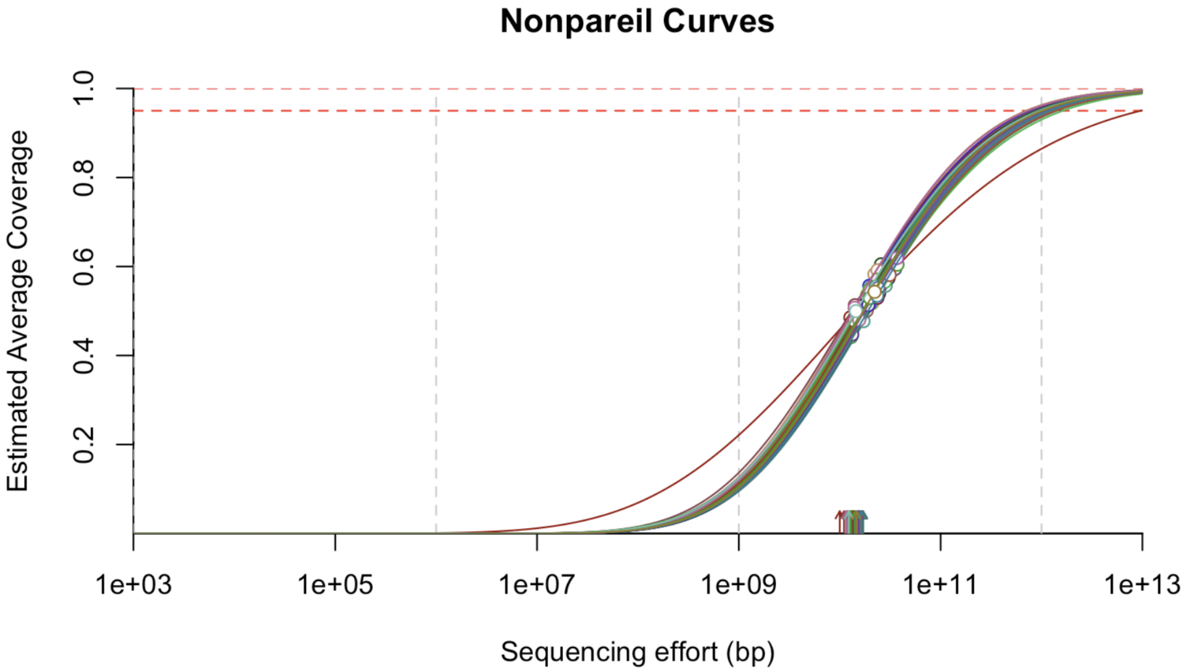

Figure S5: Fitted nonpareil curves for metagenomic samples. Empty circles indicate the actual sequencing effort and arrows the community diversity. Horizontal red dashed lines represent 95% and 99% coverage, respectively. All samples investigated had more than 45% coverage in diversity (average of 53.4 ± 3.73 %). Coverage and diversity estimations by Nonpareil are based on the redundancy of reads in metagenomic datasets; plotted curves represent the fitted model of actual (until dot) and needed sequencing effort to achieve complete coverage of diversity.

f. Most abundant microbes across all samples with full taxonomy information.

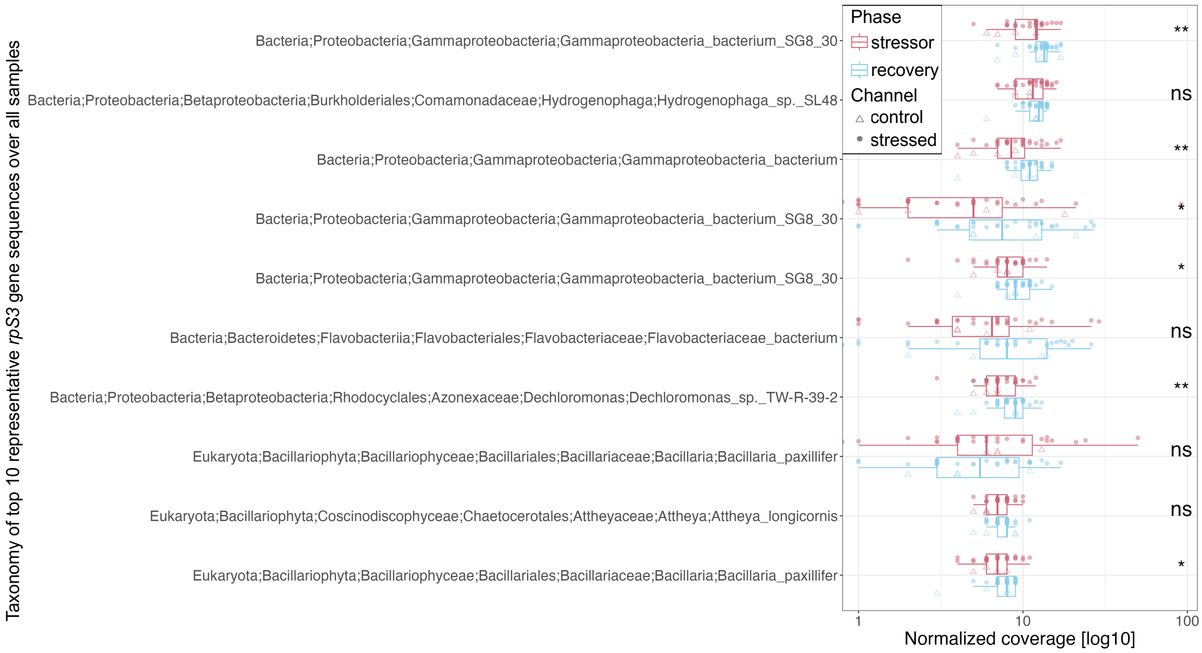

Figure S6: Most abundant microbes across all samples (ordered from top to bottom based on abundance) with full taxonomy information. Sequencing-depth normalized counts of representative *rpS3* gene sequences were summed up over all samples and taxonomy of highest ten genes was annotated. Differences between stressor and recovery phase were tested using the Wilcoxon test.

g. Species-specific stressor response based on rpS3 gene analysis.

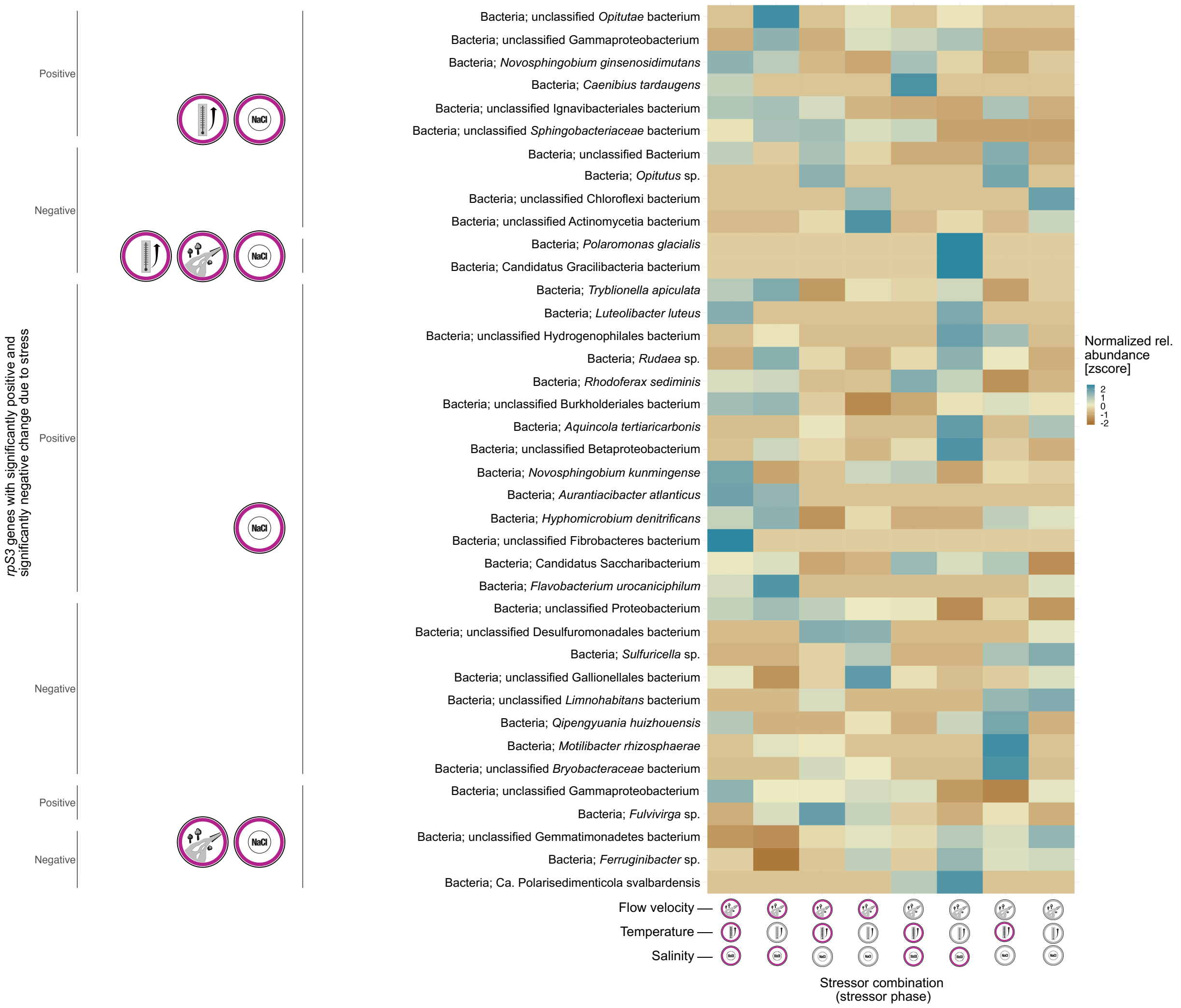

Figure S7: Microbial taxa sensitive to increased salinity based on sequencing-depth normalized representative *rpS3* gene sequences (ANOVA followed by TukeyHSD; adjusted p-value < 0.01). If multiple stressor effects per taxon were significant, only the lowest p-value was chosen. All taxa responding significantly to a stressor are summarized in Supplementary File SI6.

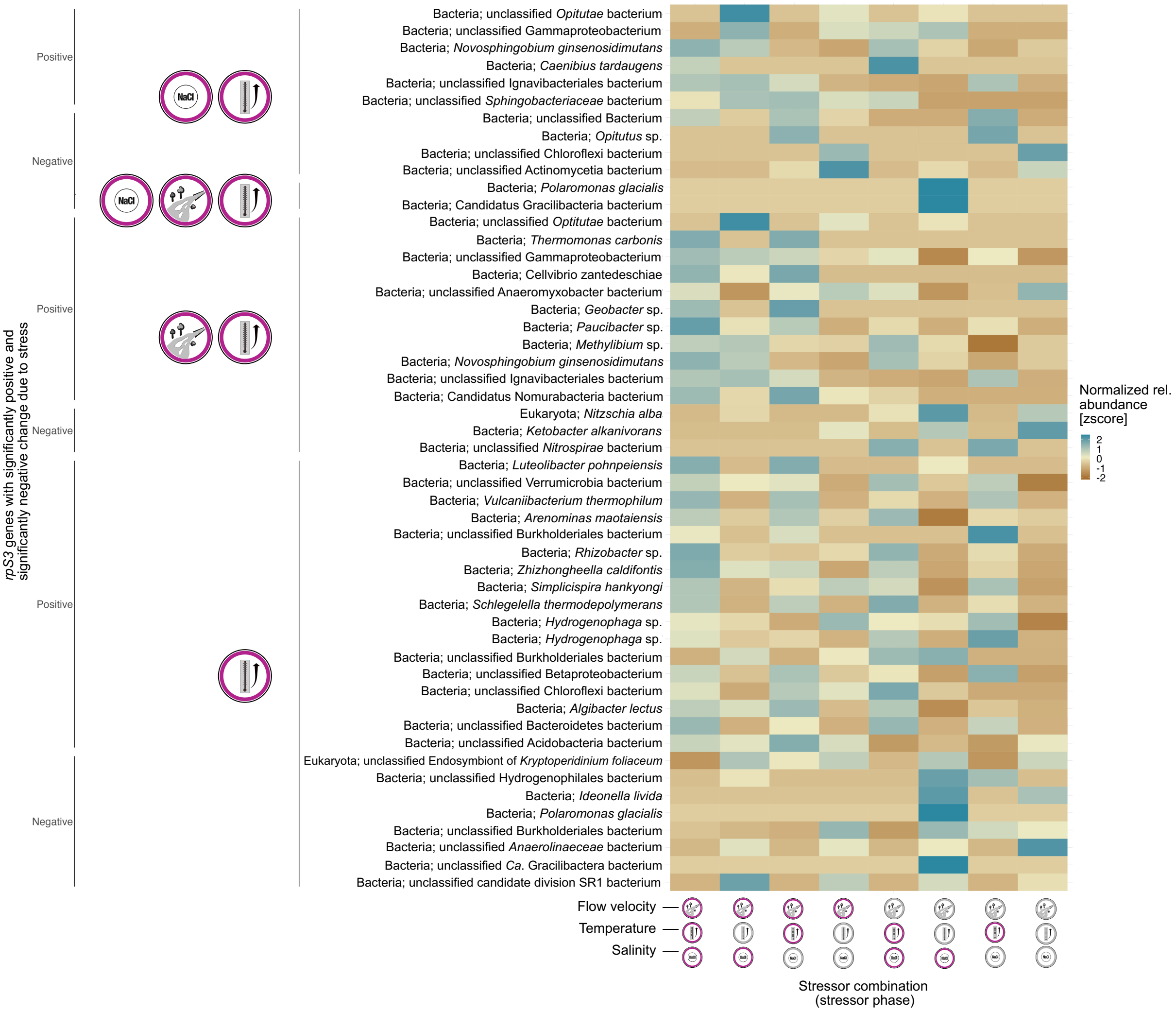

Figure S8: Microbial taxa sensitive to increased temperature based on sequencing-depth normalized representative *rpS3* gene sequences (ANOVA followed by TukeyHSD; adjusted p-value < 0.01). If multiple stressor effects per taxon were significant, only the lowest p-value was chosen. All taxa responding significantly to a stressor are summarized in Supplementary File SI6.

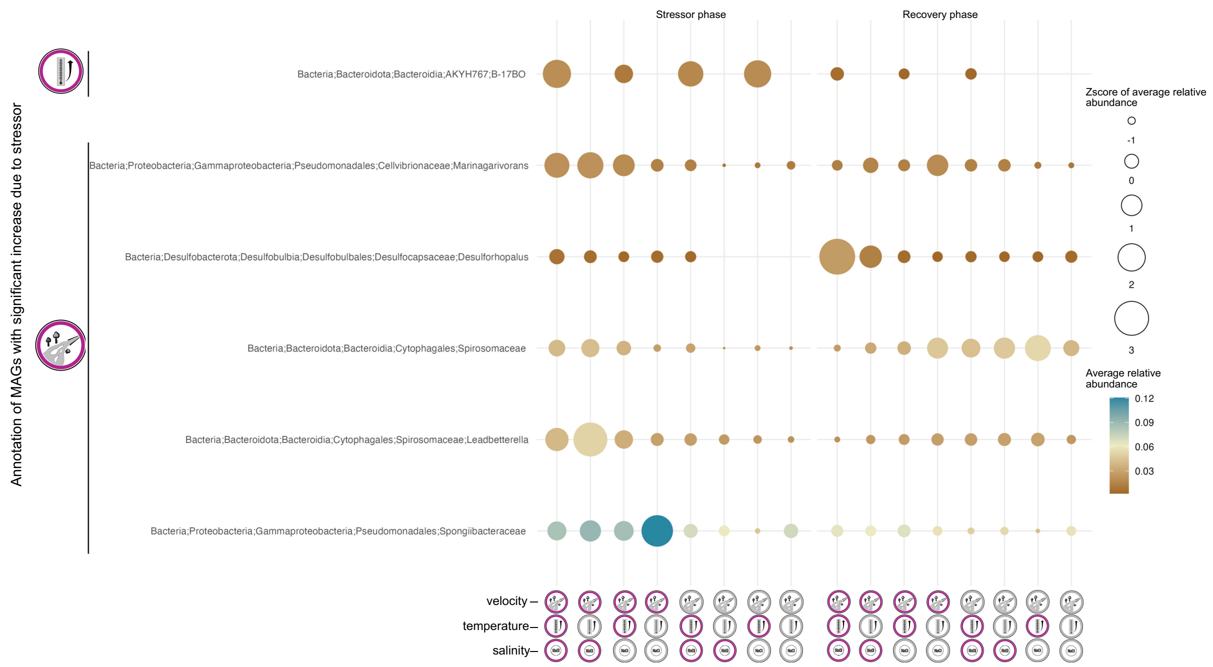

Figure S9: Relative abundance (> 0.1 %) of MAGs with significant response across all mesocosms (n=64), i.e., three MAGs responded positively to lowered flow velocity and one MAG to temperature increase. The latter one was only present in samples with increased temperature indicating a sentinel species.

h. Metatranscriptomic analysis of MAGs.

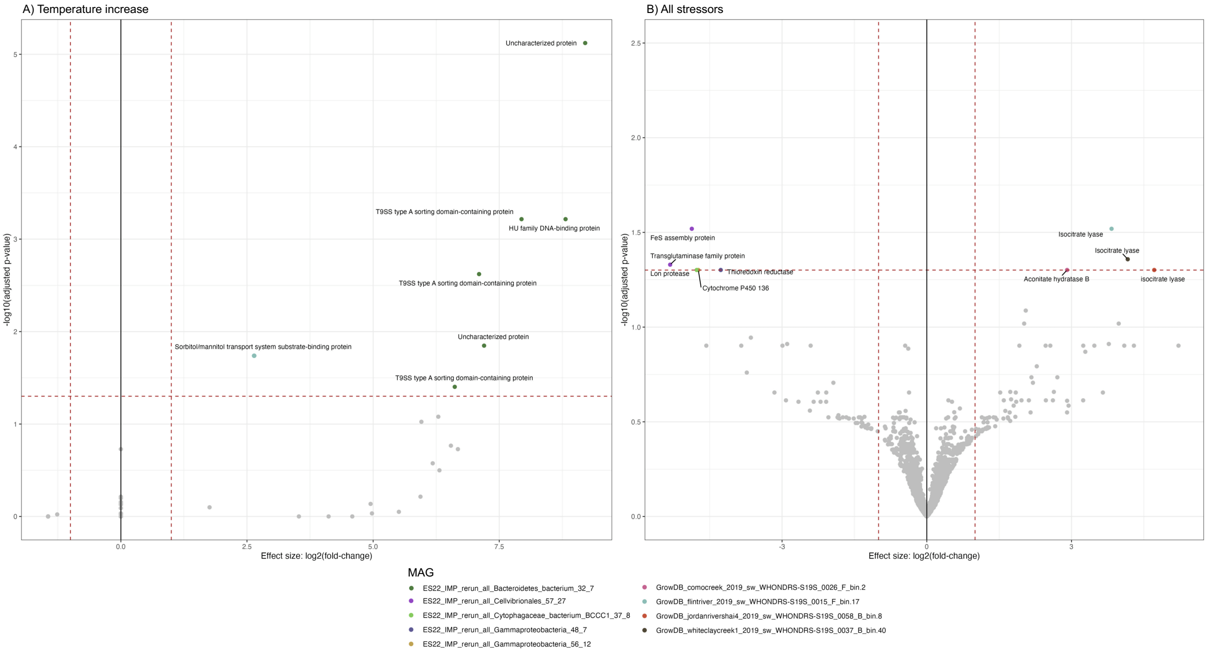

Figure S10: Metatranscriptomic reads were mapped to the set of MAGs and tested for differential expression due to temperature increase and all stressors in combination using DESeq2 (n=32 for stressor phase; log2(fold-change) > 1 and adjusted p-value < 0.05; [3]).

i. Statistical testing of encoded ecosystem functions.

Table S2: Statistical testing of stressor impact on microbial ecosystem functions from metagenomic sequencing. Counts of functional HMMs annotated to metagenomic assemblies by METABOLIC (v4.0) [4] were normalized by sequencing depth. Adonis2 was run with 999 permutations, a model including all single factors and interaction terms with strata set for stressor and recovery phase and based on the marginal effects of the terms as test design.

Descriptor	Df	SumOfSqs	R2	F	Pr(>F)
temperature	1	0.06000	0.04	2.29	0.120
salinity	1	0.03728	0.02	1.44	0.229
velocity	1	0.01748	0.01	0.67	0.421
temperature:salinity	1	0.07736	0.05	3.10	0.060
temperature:velocity	1	0.00580	0.003	0.23	0.731
salinity:velocity	1	0.06564	0.04	2.63	0.105
temperature:salinity: velocity	1	0.0521	0.03	2.13	0.133

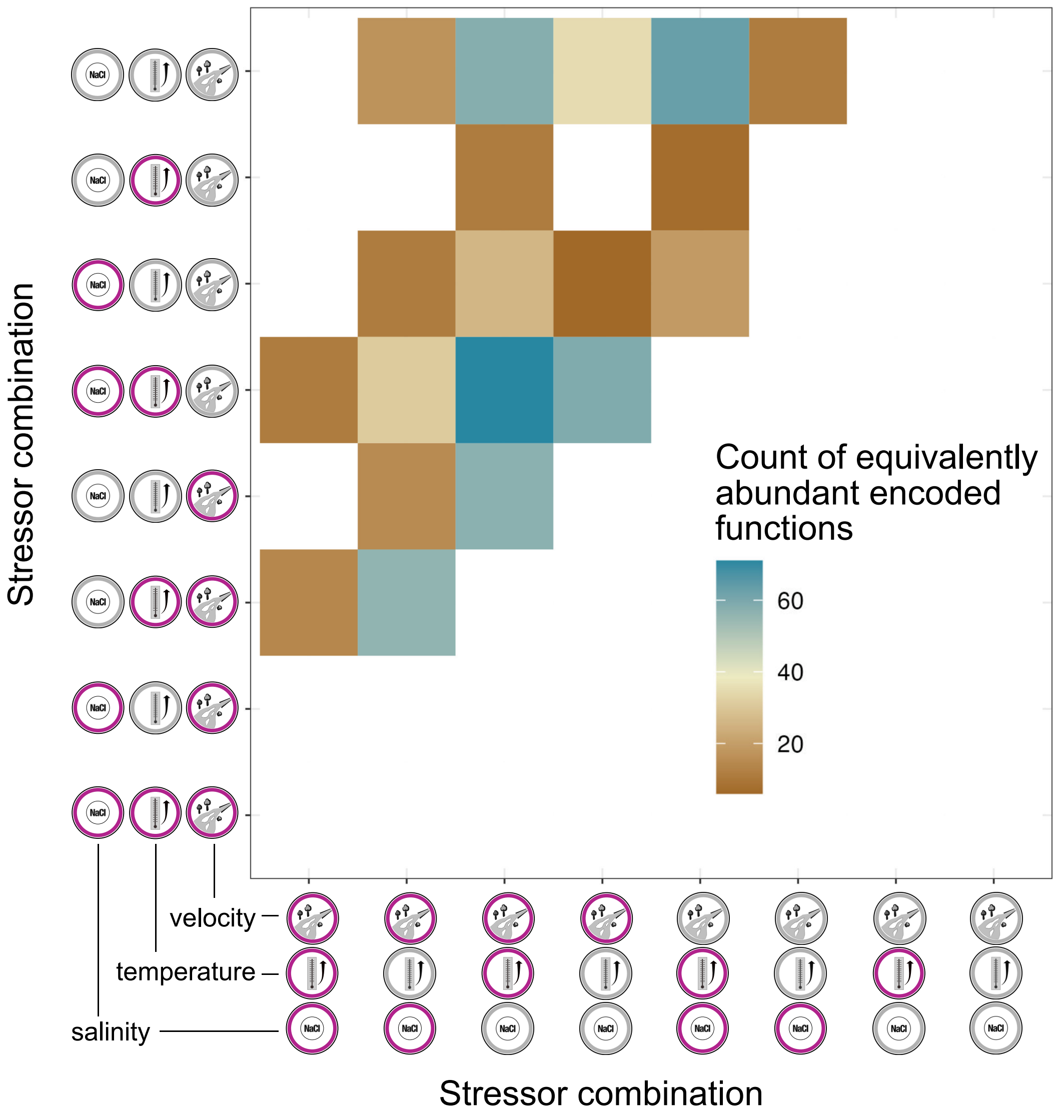

Figure S11: Functional analysis of metagenomic data. Metagenomic assemblies were tested for equivalent abundant functions. Count of significantly equivalently abundant metabolic modules (of in total 314) between the respective stressor pairs (n=32) after the stressor phase indicates that lesser functions are equally shared when more stressors are applied.

2. Supplementary materials and methods

a. Experiment design and mesocosm setup.

The *ExStream* system is an open system, *i.e.*, after passing through the experimental system the water is directed back into the stream (see [5]). In every mesocosm, 1 liter of sediment from Boye stream (0-1 mm) was added, along with 100 ml slurry of fine particulate organic matter (from a small tributary stream close to the Boye stream; 51.5627885 °N, 6.9150225 °E), serving as an initial carbon source in order to represent the typical substrate of the studied river [5]. Gravel (quartz stones, 6-8 mm, store-bought) was positioned behind the inflow jet with three pebbles (quarry stones, 40-80mm, store-bought) on one side of the gravel section and another pebble (quarry stones, 32-56 mm, store-bought) on the other side. Organic matter was added to the mesocosms by using air-dried alder leaves (*Alnus glutinosa*) collected from the coordinates 51°20′59.09″N, 7°10′14.03″E. Water temperature was constantly measured in 8 representative channels using data loggers, conductivity and water temperature was measured separately using external probes (see Supplementary File SI12). The leaves were packed into fine mesh bags before being added to the mesocosms. A check for potential prokaryotic contamination of the mesocosms based on full-length 16S rRNA gene Nanopore sequencing was done beforehand showing no detectable contamination (see below).

b. Contamination assessment of ExStream mesocosm system.

Three single ExStream channels were tested for contamination by filtering circulated flow-through water followed by DNA extraction and Nanopore 16S rRNA gene sequencing. The outflow of an empty channel was connected with a filter system (0.1 mm pore size, JVWP14225) where filtered water was fed again into the mesocosm. This setup was run for nine hours with 10 liter ultrapure water. After cleaning the piping system with diluted bleach, the check was repeated two times with different mesocosm channels. Extraction of DNA was performed based on the DNeasy PowerMax Soil Kit (Qiagen, Germany) with overnight precipitation in 70% EtOH and glycogen as the carrier. Although extracted DNA amounts were too low, 16S rRNA gene sequencing (16S Barcoding Kit 1-24 SQK-16S024, Nanopore) was performed with maximum DNA input resulting in under 30 reads per sample. With working positive and negative controls, no biological contamination was detectable.

c. Temperature as stressor.

Temperature increase in mesocosm was introduced via heated water from an electric mobile heating system (triMobil EHZ36, maximum capacity: 36 kW) using filtered (125 µm stainless steel filter that was automatically flushed every 10 min for 15 s) stream water as input. The heated water was drained into 10 L buckets installed above the header tank. Heated water was mixed with cold water from the stream to achieve +3.45 °C (treatment means ± SD: $T_{ambient}$

= 8.71 ± 0.06 °C, $T_{warming}$ = 12.16 ± 0.08 °C, n = 4) degree water in the mesocosm via a static mixer [6]. Header tanks that received only unheated stream water were instead supplied with an amount of filtered stream water that was equivalent to the heated water.

d. Salinity as stressor.

Salt tablets (Claramat, > 99.9 % NaCl) mixed with stream water were supplied through dosage pumps to mesocosm via dripper lines to receive an increase by 0.529 mS/cm (treatment means ± SD: $EC_{ambient}$ = 0.842 ± 0.006 mS/cm, EC_{salt} = 1.343 ± 0.151 mS/cm, n=32), corresponding to 154.1 mg/L added chloride. The salinity setup resembled the methodology outlined in the [7].

e. Velocity as stressor.

The velocity of water was lowered by removing the inflow jet [5]. The lowered flow mesocosms had 3.50 ± 3.32 cm/s (n = 4, measured in the middle of the water column, in the first quarter of a mesocosm) and the normal flow velocity with an inflow jet mounted had 14.25 ± 7.59 cm/s (n = 4). The normal flow velocity corresponded to a typical flow velocity of the studied stream type, *i.e.*, a small sand-dominated lowland river (type 14 according to [8]). Consequently, the lowered flow velocity was designed to be outside of the normal flow velocity of the model river type.

f. Deviation from the number of replicates and offset of stressors.

Due to an oversight during initial setup, the number of replicates differed for four stressor combinations., *i.e.*, N=3 for V-S+T+ (velocity lowered, salinity increased, temperature increased) and V0S0T+, and N=5 for V-S0T+ and V0S+T+ during the stressor phase. Unpredictable, yet natural, fluctuations in streamflow and water level occurred between days 7 and 10 of the stressor phase. These fluctuations, caused by heavy rainfall in the catchment area, resulted in temporary pump blockages. Consequently, stressors were only applied for a limited period (12 hours) on day 8. Normal conditions resumed on the morning of day 10, allowing for the continuation of the stressor phase as planned.

g. DNA and RNA extraction and sequencing.

DNA was extracted for metagenomic and 16S rRNA gene amplicon sequencing from 0.5 g sediment input. For DNA extraction, samples were mixed with 0.1 and 0.5-mm diameter glass beads and 100 µl Proteinase K, 5 µl RNAse A, and 900 µl TNES (for buffer and reagents see materials in [9]. Then, samples were bead-beaten for 2 min at 2400 rpm in a Mini-Bead-Beater 96 (Biospec Products, Bartlesville, USA). Samples were incubated at 56 °C and bead beaten at 1400 rpm for 20 minutes. Lysates were divided for replicates and DNA was extracted

following the spin column protocol using a vacuum manifold described in [10]. DNA clean-up was performed with carboxylated-magnetic beads and PEG-NaCl buffer following the protocol described in [11] with 40 µl DNA input and 80 µl of clean-up solution. Eluted DNA was split and used for metagenomic sequencing directly or library preparation for 16S rRNA gene amplicon sequencing. RNA was extracted from sediment for metatranscriptomic sequencing using phenol/chloroform/isoamyl alcohol as described in [12]. Negative controls for DNA and RNA extraction were successful.

h. Transcriptomic RNA sequencing and read processing.

Total RNA extracts were sequenced using the Illumina TruSeq Stranded Total RNA with Ribo-Zero PLUS Kit for rRNA depletion. Raw metatranscriptomic sequences were processed with a custom snakemake-based workflow [13] (https://github.com/adeep619/Vasuki). In this workflow, raw sequences were quality filtered using cutadapt (v3.2, [14]) with phred score 20 and minimum length 50 bp. Ribosomal RNA sequences were removed from the quality checked and adapter-trimmed sequences using Ribodetector [15].

i. 16S rRNA gene amplicon analysis.

For 16S rRNA gene analysis, DNA was amplified with a two-step PCR approach. 10 µl reaction volume was used per sample for the first PCR with Multiplex PCR Plus Kit (Qiagen) with primers 515f/806r [16], and 1 µl of DNA input. After the cleanup of the first PCR product [11], 2 µl of DNA was used for the second PCR. Cycling conditions for the first and second PCR are given in Supplementary Table S3. Bead-based normalization protocol was used for DNA normalization which resulted in a 2 ng/µl concentration [17]. The pooled libraries were concentrated using a spin-column clean-up protocol with a final volume of 100 µL [10]. PCR replicates were produced from each sample and these libraries were then subjected to paired-end sequencing (2 x 250 bp) on Illumina NovaSeq (CeGat Gmbh, Tübingen).

Table S3: Cycling conditions for 16S rRNA gene amplification. The first PCR was run for efficient amplification using the target group primers while the second step was performed for individual tagging of the samples and addition of the Illumina adapters. Per sample a reaction volume of 10 µl was used. For 1st PCR: 5 µl Multiplex Master Mix, 0.2 µl forward/reverse primer, 1 µl sample DNA, 3.6 µl water. For 2nd PCR: 5 µl Multiplex Master Mix, 1 µl Coral Load, 1 µl forward/reverse primer, 2 µl sample DNA.

PCR phase	Time	1st PCR-Temperature [°C] 20 cycles	2nd PCR-Temperature [°C] 25 cycles
Initial denaturation	5 min	95	95
Denaturation (25 cycles)	30 s	95	95
Annealing (25 cycles)	90 s	50	61
Elongation (25 cycles)	30 s	72	72
Final elongation	10 min	68	68

Sequenced 16S rRNA gene amplicons were processed with the Natrix2 workflow [18]. In brief, the pipeline included primer removal, assembly with Pandaseq (v2.11, [19]) and filtering the paired-end reads with a alignment threshold score of 0.9 and sequence length with a minimum of 100 bp and a maximum of 600 bp. In the case of ASVs, primers were removed with Cutadapt [14] and denoising was performed with DADA2 (v1.30.0, [20]), for both ASVs and OTUs, dereplication (100% sequence similarity) and removal of chimeric sequences was done with cd-hit (v 4.8.1, [21]); erroneous sequences were removed with a split sample approach using AmpliconDuo (v1.1, [22]). Resulting sequences were clustered with Swarm (v2.2.2, [23]) into OTUs and ASVs which were aligned against the Silva database (v138.1, [24]) by using Mothur (v1.40.5, [25]) for taxonomic classification. MUMU (https://github.com/frederic-mahe/mumu), an update for LULU [26] was used for post-clustering for OTUs. For quality assessment, PCR replicates of each sample were consolidated by eliminating OTUs exclusive to one of the replicates and combining the reads from the remaining replicates. Additionally, number of reads for OTUs identified in negative controls were subtracted from all individual samples (46 OTUs).

j. Metagenomic sequencing and quality control.

Metagenomic sequencing was performed using the Illumina DNA PCR-Free Prep protocol on a NovaSeq 6000 with a minimum sequencing depth of 30 Gbp (150 bp paired-end reads; CeGat Gmbh, Tübingen). Metagenomic reads were quality checked and trimmed using BBduk (Bushnell, https://jgi.doe.gov/data-and-tools/bbtools/bb-tools-user-guide/) and Sickle (quality

score ≥ 20 and minimum read length ≥ 20 bp) [27]. The covered microbial diversity by metagenomic sequences was estimated using Nonpareil3 (v3.4.1; kmer mode) [2].

k. Ribosomal protein S3 marker gene analysis.

Positive hits for *rpS3* genes from both described approaches, *i.e.,* from HMMs and FunTaxDB, were searched against a *rpS3* set for archaea and bacteria from GTDB r207 [28] using USEARCH [29] reporting the best hit with an e-value of 1E-5 or better (or reporting unclassified if no hit with these criteria was found).

For statistical analyses of *rpS3* gene sequences across samples, *rpS3* gene sequences were clustered using USEARCH (-cluster_fast -id 0.99). Per cluster, the centroid of the cluster was chosen as the representative sequence if flanked by at least 1 kb in both directions on its contig. Otherwise, a non-centroid longest sequence that was respectively flanked could be extended or just the longest sequence was selected. The contigs were then trimmed to the respective length including the flanking regions in order to improve mapping results [30]. Then, quality-filtered reads were mapped with bowtie2 [31] representative *rpS3* gene sequences excluding reads shorter than 100 bp (reduction of 3.1% ± 0.29% on sequence length) to ensure a sensitive and specific mapping. Mappings were filtered for three mismatches or less to exclude random alignments and coverage was used for statistical analysis.

The mean dissimilarity between all treatments was calculated, tested using the wilcox test, and resulting p-values were adjusted using the Bonferroni method. Based on the dissimilarity matrix, nonmetric multidimensional scaling (NMDS) with grouping per stressor at a level of 0.75 and Multi-Response Permutation Procedure (MRPP) tests were performed using the vegan package in R [32].

Additionally, each representative sequence was tested for stressor effects after the stressor phase only. For that, mapping-based relative abundances were normalized based on the lowest sequencing depth, and each sequence was tested as follows. A two-way anova was performed and the resulting p-value was adjusted for multiple testing using the Bonferroni method. Subsequently, for representative sequences with a p-value lower than 0.05, a post hoc Tukey-HSD test was run.

l. Binning of assembled metagenomes into MAGs and annotation.

MetaBAT2 [33], ABAWACA [34], and MaxBin2 [35] were used as binning tools. For the latter, a cross-mapping over all samples was done using Bowtie2 [31], and both marker sets were used. In the end, an optimized set of bins per sample was produced using DASTool [36], and resulting MAGs were dereplicated using dRep v3.4.3 [37]. For that, Checkm2 [38] was run for all MAGs and the output was used for genome statistic calculation by dRep with a minimum

completeness of 75% and identity of secondary clusters of 95%. Contamination threshold was not set since resulting MAGs were afterwards manually curated using uBin v0.9.20 [39].
The final set of MAGs was taxonomically annotated using GTDB-Tk (v2.1.0, r207) workflow *de_novo* and taxonomic classifications were verified via the *classify_wf* mode [40]. Plotting of phylogenetic tree was performed with the help of ggplot2 [41], ggdendro [42], ggtree [43], ggtreeExtra [44], rcartocolor [45] and patchwork [46]. Functional annotation of the final MAG set was performed using DRAM (v1.4.6; [47]) and final quality assessment of MAGs according to [48] was done as described in [49]. A detailed annotation for our keystone MAG (Marinilabiliales_41_12) was done in MicroScope [50]. For this MAG, a detailed transcriptomic analysis was done based on the annotation from MicroScope, by mapping the transcriptomic reads using Bowtie2 [31] and counting hits using featureCounts (v1.5.3) [51].

j. Functional annotation and equivalence testing.

Metabolic pathways were annotated to the metagenomic assemblies based on METABOLIC (v4.0) [4] using the METABOLIC-G.pl mode. Relative abundance of HMM-modules was calculated by the mean coverage of contigs which encoded the respective genes and normalized on sequencing depth. Response to stressors was tested using multivariate statistics including adonis2 [32] and stressor combinations were tested for equivalent abundance of functional modules using the TOSTER package in R [52]. Taking the given number of mesocosms per treatment, the higher and lower equivalence bounds were calculated to achieve 33% power as suggested previously [53].

3. References

1. Kassambara A. ggpubr: ‘ggplot2’ Based Publication Ready Plots. 2023.
2. Rodriguez-R LM, Gunturu S, Tiedje JM, Cole JR, Konstantinidis KT. Nonpareil 3: Fast Estimation of Metagenomic Coverage and Sequence Diversity. *mSystems* 2018;**3**:e00039-18. https://doi.org/10.1128/mSystems.00039-18
3. Love MI, Huber W, Anders S. Moderated estimation of fold change and dispersion for RNA-seq data with DESeq2. *Genome Biol* 2014;**15**:550. https://doi.org/10.1186/s13059-014-0550-8
4. Zhou Z, Tran PQ, Breister AM, Liu Y, Kieft K, Cowley ES, et al. METABOLIC: high-throughput profiling of microbial genomes for functional traits, metabolism, biogeochemistry, and community-scale functional networks. *Microbiome* 2022;**10**:33. https://doi.org/10.1186/s40168-021-01213-8
5. Madge Pimentel I, Baikova D, Buchner D, Burfeid Castellanos A, David GM, Deep A, et al. Assessing the response of an urban stream ecosystem to salinization under different flow regimes. *Science of The Total Environment* 2024;**926**:171849. https://doi.org/10.1016/j.scitotenv.2024.171849
6. Madge Pimentel I, Rehsen PM, Beermann AJ, Leese F, Piggott JJ, Schmuck S. An automated modular heating solution for experimental flow-through stream mesocosm systems. *Limnology & Ocean Methods* 2023;lom3.10596. https://doi.org/10.1002/lom3.10596
7. David GM, Pimentel IM, Rehsen PM, Vermiert A-M, Leese F, Gessner MO. Multiple stressors affecting microbial decomposer and litter decomposition in restored urban streams: Assessing effects of salinization, increased temperature, and reduced flow velocity in a field mesocosm experiment. *Science of The Total Environment* 2024;**943**:173669. https://doi.org/10.1016/j.scitotenv.2024.173669
8. Dahm V, Kupilas B, Rolauffs P, Hering D, Haase P, Kappes H, et al. Strategien zur Optimierung von Fließgewässer-Renaturierungsmaßnahmen und ihrer Erfolgskontrolle. Umweltbundesamt, 2014.
9. Buchner D. Co-extraction of RNA and DNA from soil and sediment samples v1. 2022.
10. Buchner D. Guanidine-based DNA extraction with silica-coated beads or silica spin columns v1. 2022.
11. Buchner D. PCR cleanup and size selection with magnetic beads v2. 2022.
12. Buchner D. Sample preparation and lysis of homogenized malaise trap samples v1. 2022.
13. Köster J, Rahmann S. Snakemake—a scalable bioinformatics workflow engine. *Bioinformatics* 2012;**28**:2520–2522. https://doi.org/10.1093/bioinformatics/bts480
14. Martin M. Cutadapt removes adapter sequences from high-throughput sequencing reads. *EMBnet j* 2011;**17**:10. https://doi.org/10.14806/ej.17.1.200
15. Deng Z-L, Münch PC, Mreches R, McHardy AC. Rapid and accurate identification of ribosomal RNA sequences via deep learning. *Nucleic Acids Research* 2022;**50**:e60–e60. https://doi.org/10.1093/nar/gkac112
16. Apprill A, McNally S, Parsons R, Weber L. Minor revision to V4 region SSU rRNA 806R gene primer greatly increases detection of SAR11 bacterioplankton. *Aquat Microb Ecol* 2015;**75**:129–137. https://doi.org/10.3354/ame01753
17. Buchner D. PCR normalization and size selection with magnetic beads v1. 2022.
18. Deep A, Bludau D, Welzel M, Clemens S, Heider D, Boenigk J, et al. Natrix2 – Improved amplicon workflow with novel Oxford Nanopore Technologies support and enhancements in clustering, classification and taxonomic databases. *MBMG* 2023;**7**:e109389. https://doi.org/10.3897/mbmg.7.109389
19. Masella AP, Bartram AK, Truszkowski JM, Brown DG, Neufeld JD. PANDAseq: paired-end assembler for illumina sequences. *BMC Bioinformatics* 2012;**13**:31. https://doi.org/10.1186/1471-2105-13-31
20. Callahan BJ, McMurdie PJ, Rosen MJ, Han AW, Johnson AJA, Holmes SP. DADA2: High-resolution sample inference from Illumina amplicon data. *Nat Methods* 2016;**13**:581–583. https://doi.org/10.1038/nmeth.3869
21. Fu L, Niu B, Zhu Z, Wu S, Li W. CD-HIT: accelerated for clustering the next-generation sequencing data. *Bioinformatics* 2012;**28**:3150–3152.

https://doi.org/10.1093/bioinformatics/bts565
22. Lange A, Jost S, Heider D, Bock C, Budeus B, Schilling E, et al. AmpliconDuo: A Split-Sample Filtering Protocol for High-Throughput Amplicon Sequencing of Microbial Communities. *PLoS ONE* 2015;**10**:e0141590. https://doi.org/10.1371/journal.pone.0141590
23. Mahé F, Rognes T, Quince C, De Vargas C, Dunthorn M. Swarm: robust and fast clustering method for amplicon-based studies. *PeerJ* 2014;**2**:e593. https://doi.org/10.7717/peerj.593
24. Quast C, Pruesse E, Yilmaz P, Gerken J, Schweer T, Yarza P, et al. The SILVA ribosomal RNA gene database project: improved data processing and web-based tools. *Nucleic Acids Research* 2012;**41**:D590–D596. https://doi.org/10.1093/nar/gks1219
25. Schloss PD, Westcott SL, Ryabin T, Hall JR, Hartmann M, Hollister EB, et al. Introducing mothur: Open-Source, Platform-Independent, Community-Supported Software for Describing and Comparing Microbial Communities. *Appl Environ Microbiol* 2009;**75**:7537–7541. https://doi.org/10.1128/AEM.01541-09
26. Frøslev TG, Kjøller R, Bruun HH, Ejrnæs R, Brunbjerg AK, Pietroni C, et al. Algorithm for post-clustering curation of DNA amplicon data yields reliable biodiversity estimates. *Nat Commun* 2017;**8**:1188. https://doi.org/10.1038/s41467-017-01312-x
27. Joshi N, Fass J. Sickle: A sliding-window, adaptive, quality-based trimming tool for FastQ files. 2011. 2011.
28. Parks DH, Chuvochina M, Rinke C, Mussig AJ, Chaumeil P-A, Hugenholtz P. GTDB: an ongoing census of bacterial and archaeal diversity through a phylogenetically consistent, rank normalized and complete genome-based taxonomy. *Nucleic Acids Research* 2022;**50**:D785–D794. https://doi.org/10.1093/nar/gkab776
29. Edgar RC. Search and clustering orders of magnitude faster than BLAST. *Bioinformatics* 2010;**26**:2460–2461. https://doi.org/10.1093/bioinformatics/btq461
30. Figueroa-Gonzalez PA, Bornemann TLV, Adam PS, Plewka J, Révész F, Von Hagen CA, et al. Saccharibacteria as Organic Carbon Sinks in Hydrocarbon-Fueled Communities. *Front Microbiol* 2020;**11**:587782. https://doi.org/10.3389/fmicb.2020.587782
31. Langmead B, Salzberg SL. Fast gapped-read alignment with Bowtie 2. *Nat Methods* 2012;**9**:357–359. https://doi.org/10.1038/nmeth.1923
32. Oksanen J, Blanchet FG, Kindt R, Legendre P, Minchin P, O'Hara R, et al. Vegan: Community Ecology Package. R Package Version. 2.0-10. *CRAN* 2013.
33. Kang DD, Li F, Kirton E, Thomas A, Egan R, An H, et al. MetaBAT 2: an adaptive binning algorithm for robust and efficient genome reconstruction from metagenome assemblies. *PeerJ* 2019;**7**:e7359. https://doi.org/10.7717/peerj.7359
34. Brown CT, Hug LA, Thomas BC, Sharon I, Castelle CJ, Singh A, et al. Unusual biology across a group comprising more than 15% of domain Bacteria. *Nature* 2015;**523**:208–211. https://doi.org/10.1038/nature14486
35. Wu Y-W, Simmons BA, Singer SW. MaxBin 2.0: an automated binning algorithm to recover genomes from multiple metagenomic datasets. *Bioinformatics* 2016;**32**:605–607. https://doi.org/10.1093/bioinformatics/btv638
36. Sieber CMK, Probst AJ, Sharrar A, Thomas BC, Hess M, Tringe SG, et al. Recovery of genomes from metagenomes via a dereplication, aggregation and scoring strategy. *Nature Microbiology* 2018;**3**:836–843. https://doi.org/10.1038/s41564-018-0171-1
37. Olm MR, Brown CT, Brooks B, Banfield JF. dRep: a tool for fast and accurate genomic comparisons that enables improved genome recovery from metagenomes through de-replication. *ISME J* 2017;**11**:2864–2868. https://doi.org/10.1038/ismej.2017.126
38. Chklovski A, Parks DH, Woodcroft BJ, Tyson GW. CheckM2: a rapid, scalable and accurate tool for assessing microbial genome quality using machine learning. *Nat Methods* 2023;**20**:1203–1212. https://doi.org/10.1038/s41592-023-01940-w
39. Bornemann TLV, Adam PS, Turzynski V, Schreiber U, Figueroa-Gonzalez PA, Rahlff J, et al. Genetic diversity in terrestrial subsurface ecosystems impacted by geological degassing. *Nat Commun* 2022;**13**:284. https://doi.org/10.1038/s41467-021-27783-7
40. Chaumeil P-A, Mussig AJ, Hugenholtz P, Parks DH. GTDB-Tk: a toolkit to classify genomes with the Genome Taxonomy Database. *Bioinformatics* 2020;**36**:1925–1927. https://doi.org/10.1093/bioinformatics/btz848
41. Wickham H. ggplot2: elegant graphics for data analysis, Second edition. Switzerland:

Springer, 2016.
42. Vries A de, Ripley BD. ggdendro: Create Dendrograms and Tree Diagrams Using ‘ggplot2’. 2022.
43. Yu G. Using ggtree to Visualize Data on Tree-Like Structures. *Curr Protoc Bioinformatics* 2020;**69**:e96. https://doi.org/10.1002/cpbi.96
44. Xu S, Dai Z, Guo P, Fu X, Liu S, Zhou L, et al. ggtreeExtra: Compact Visualization of Richly Annotated Phylogenetic Data. *Molecular Biology and Evolution* 2021;**38**:4039–4042. https://doi.org/10.1093/molbev/msab166
45. Nowosad J. ‘CARTOColors’ Palettes. 2018.
46. Pedersen TL. patchwork: The Composer of Plots. 2023.
47. Shaffer M, Borton MA, McGivern BB, Zayed AA, La Rosa SL, Solden LM, et al. DRAM for distilling microbial metabolism to automate the curation of microbiome function. *Nucleic Acids Research* 2020;**48**:8883–8900. https://doi.org/10.1093/nar/gkaa621
48. Bowers RM, Kyrpides NC, Stepanauskas R, Harmon-Smith M, Doud D, Reddy TBK, et al. Minimum information about a single amplified genome (MISAG) and a metagenome-assembled genome (MIMAG) of bacteria and archaea. *Nature Biotechnology* 2017;**35**:725–731. https://doi.org/10.1038/nbt.3893
49. Stach TL, Sieber G, Shah M, Simon SA, Soares A, Bornemann TLV, et al. Temporal disturbance of a model stream ecosystem by high microbial diversity from treated wastewater. *MicrobiologyOpen* 2023;**12**:e1347. https://doi.org/10.1002/mbo3.1347
50. Vallenet D, Engelen S, Mornico D, Cruveiller S, Fleury L, Lajus A, et al. MicroScope: a platform for microbial genome annotation and comparative genomics. *Database* 2009;**2009**. https://doi.org/10.1093/database/bap021
51. Liao Y, Smyth GK, Shi W. featureCounts: an efficient general purpose program for assigning sequence reads to genomic features. *Bioinformatics* 2014;**30**:923–930. https://doi.org/10.1093/bioinformatics/btt656
52. Lakens D. Equivalence Tests: A Practical Primer for *t* Tests, Correlations, and Meta-Analyses. *Social Psychological and Personality Science* 2017;**8**:355–362. https://doi.org/10.1177/1948550617697177
53. Simonsohn U. Small Telescopes: Detectability and the Evaluation of Replication Results. *Psychol Sci* 2015;**26**:559–569. https://doi.org/10.1177/0956797614567341
